# Transcriptomic Analysis for Different Sex Types of *Ricinus communis* L. during Development from Apical Buds to Inflorescences by Digital Gene Expression Profiling

**DOI:** 10.3389/fpls.2015.01208

**Published:** 2016-02-12

**Authors:** Meilian Tan, Jianfeng Xue, Lei Wang, Jiaxiang Huang, Chunling Fu, Xingchu Yan

**Affiliations:** ^1^Key Laboratory of Biology and Genetic Improvement of Oil Crops, Ministry of Agriculture, Oil Crops Research Institute of the Chinese Academy of Agricultural SciencesWuhan, China; ^2^Castor Oil Research Institute of JiaxiangZibo, China

**Keywords:** castor bean, digital expression profile, pistillate, sex determination, transcriptome analysis

## Abstract

The castor plant (*Ricinus communis* L.) is a versatile industrial oilseed crop with a diversity of sex patterns, its hybrid breeding for improving yield and high purity is still hampered by genetic instability of female and poor knowledge of sex expression mechanisms. To obtain some hints involved in sex expression and provide the basis for further insight into the molecular mechanisms of castor plant sex determination, we performed DGE analysis to investigate differences between the transcriptomes of apices and racemes derived from female (JXBM0705P) and monoecious (JXBM0705M) lines. A total of 18 DGE libraries were constructed from the apices and racemes of a wild monoecious line and its isogenic female derivative at three stages of apex development, in triplicate. Approximately 5.7 million clean tags per library were generated and mapped to the reference castor genome. Transcriptomic analysis showed that identical dynamic changes of gene expression were indicated in monoecious and female apical bud during its development from vegetation to reproduction, with more genes expressed at the raceme formation and infant raceme stages compare to the early leaf bud stage. More than 3000 of differentially expressed genes (DEGs) were detected in *Ricinus* apices at three developmental stages between two different sex types. A number of DEGs involved in hormone response and biosynthesis, such as auxin response and transport, transcription factors, signal transduction, histone demethylation/methylation, programmed cell death, and pollination, putatively associated with sex expression and reproduction were discovered, and the selected DEGs showed consistent expression between qRT-PCR validation and the DGE patterns. Most of those DEGs were suppressed at the early leaf stage in buds of the mutant, but then activated at the following transition stage (5-7-leaf stage) of buds in the mutant, and ultimately, the number of up-regulated DEGs was equal to that of down-regulation in the small raceme of the mutant. In this study, a large number of DEGs and some suggestions involved in sex expression and reproduction were discovered using DGE analysis, which provides large information and valuable hints for next insights into the molecular mechanism of sex determination. It is useful for other further studies in *Ricinus*.

## Introduction

Castor (*Ricinus communis* L.), an important industrial oilseed crop belonging to the Euphorbiaceae family, grows as an indeterminate annual or perennial depending on climate and soil types in tropical, sub-tropical, and warm temperate regions (Anjani, 2012). Because of the high fatty acid content in its seeds (more than 45%) and the rich ricinoleic acid content in its oil (80–90%), castor is a versatile raw material in industrial chemistry: for example, castor oil can be used for the production of lubricants, nylon, hydraulic and brake fluids, paints, dyes, coatings, inks, cold resistant plastics, waxes and polishes, pharmaceuticals, perfumes, and biodiesel (Jeong and Park, 2009; Halilu et al., 2013). To date, several high-yield varieties and hybrids have been developed (Baldanzi and Pugliesi, 1998; Amaral, 2003; Savy Filho, 2005; Pranavi et al., 2011), but to meet the tremendous global demand for castor oil, cultivars with even higher yield and oil content are needed (Anjani, 2012). However, hybrid breeding for improved yield and high purity in *Ricinus* is still constrained by the genetic instability of females and the unknown mechanism of sex expression.

In castor plant, the standard type of inflorescence is gradient monoecious raceme (female flowers at the apex and staminate flowers on the lower portion) (Shifriss, 1960). However, a wide variation of inflorescence patterns occurs in natural cultivation, including other kinds of racemes such as strictly pistillate (bearing only female flowers), male (only staminate flowers), apically interspersed (monoecism with interspersed male flowers in the apical pistillate region), and entirely interspersed (female and male flowers uniformly interspersed) (George and Shifriss, 1967). In addition, inflorescence setting with one or a few hermaphrodite flowers occurs occasionally (Jacob, 1963). Accordingly, for castor individuals, there are several sex models, including normal monoecism, sex reversal, interspersed sexuality, and strictly female (Shifriss, 1956; Jacob and Atsmon, 1965; George and Shifriss, 1967).

In the past, scientists focused on the sexuality of *Ricinus* to interpret the inheritance and instability of sex variation. Shifriss (1956) described the sex tendency, sex patterns, inheritance and reversion of *Ricinus*, and proposed a hypothesis: Gene *F* controls a genetically stable series of sex variants ranging from female (*f*) to strongly male inbreds, and an unknown factor could affect male tendency, sex reversion, and sex instability by suppressing gene *F* or mutating itself. Later, he described sex variations of *Ricinus* in two genetic systems, which he tentatively named as “conventional” and “unconventional” (Shifriss, 1960). Monoecious variants and rare recessive female mutants were ascribed to the conventional form, sex reversals and non-reverted females belonged to the unconventional form. Monoecism is governed by two major groups of genes: qualitative genes that determine flower type, and polygenes regulating gradient differentiation and racial differences in sex tendency. In addition, gene modifiers such as *id* and *th* were also considered to affect the pattern of sex differentiation. He postulated that genetic instability of spontaneous mutants, so-called position-effect variegation, may result from a rearrangement undergoing two basic kinds of genetic changes: mutation into new hereditary potentialities, and transformation between an “active” and “inactive” state. Moreover, further evidence regarding the former findings (Shifriss, 1956, 1960) revealed that femaleness transmits more effectively to progeny through female inflorescences of sex-reversal plants than through reverted monoecious inflorescences of the same plant (Jacob and Atsmon, 1965). With regard to interspersed sexuality, Shifriss (1956, 1960) believed that the interspersed pattern of sex differentiation is determined by hereditary factors for femaleness and genes for interspersed staminate flowers (*id*). George and Shifriss (1967) investigated more deeply into the inheritance of interspersed inflorescence patterns, and concluded that the level of expressivity of interspersed staminate flowers depends on the dosage of two independent genes (*id1* and *id2*), their loci, and the environment.

In addition to genetic regulation, sex expression or variation of *Ricinus* is simultaneously affected to some degree by fluctuations in the environment, including temperature, vegetative activity, nutrition level, pruning, and seasonal variations (Shifriss, 1956), as well as by plant hormones (Shifriss, 1961; Philipos and Narayanaswamy, 1976; Kumar and Rao, 1980; Mohan Ram and Sett, 1980; Varkey and Nigam, 1982; Tan et al., 2011). Despite a great deal of progress, the hypotheses described above are vulnerable in the absence of cytological information, and the molecular mechanisms of sex variation and genes determining sex expression in *Ricinus* remain poorly understood.

Genome-wide analyses have dramatically improved the efficiency of gene discovery. Next-generation sequencing (NGS) technologies provide new approaches for global measurements of gene expression. Due to its high efficiency and low cost, NGS has become an attractive alternative method for more efficient study of the genome, epigenome, and transcriptome (Oshlack et al., 2010; McIntyre et al., 2011). Many plant species have benefited from this technology, and large-scale genome sequences and transcriptome data are available in both model and non-model species (Goff et al., 2002; Yu et al., 2002; Kaplan et al., 2006; Ramsey et al., 2008; Huang et al., 2009; Wang et al., 2011; Xu et al., 2013). Cucumber, as a model plant for study of floral sex expression, its sex-controlling genes, sex-modifying plant hormones, and interactions with environmental conditions are clearly understood (Galun, 1962; Trebitsh et al., 1987, 1997; Malepszy and Niemirowicz-Szczytt, 1991; Yin and Quinn, 1995; Yamasaki et al., 2000; Kater et al., 2001; Mibus and Tatlioglu, 2004; Li et al., 2009b), and a number of candidate genes required for sex determination have been identified by transcriptome profile analysis (Guo et al., 2010; Wu et al., 2010). In *Ricinus*, however, a gap still exists between interpreting the molecular mechanism of sex expression and isolating the sex-determining genes. The complete sequencing of the castor bean genome (Chan et al., 2010) provides tremendous opportunities for genomic analysis and facilitates identification of genes of biological interest. Digital gene expression (DGE) analysis, a powerful and recently developed tool, is based on ultra-high-throughput sequencing of millions of signatures in the genome, allowing identification of specific genes and direct quantitation of transcript abundance (Bentley, 2006; Hong et al., 2011). DGE analysis detects gene expression more quantitatively than microarray assays ('t Hoen et al., 2008) while providing similar assessments of relative transcript abundance. In comparison to high-throughput mRNA sequencing (RNA-seq), DGE more accurately detects expression differences in poorly expressed genes and exhibits much less transcript length bias (Hong et al., 2011). Thus, DGE has been widely utilized to discriminate differences in transcriptional responses among different tissues and organs in plants in the contexts of biotic and abiotic stress, metabolite biosynthesis, and developmental biology (Tian et al., 2013; Wei et al., 2013; Zhang et al., 2013; Guo et al., 2014; Yu et al., 2014; Zhao et al., 2014).

In this study, we performed DGE using the Illumina HiSeq 2000 to investigate the differences between the transcriptomes of apices and racemes from female (JXBM0705P) and monoecism (JXBM0705M) of *Ricinus*. In an attempt to get some hints associated with sex expression in *Ricinus*, we conducted (after high-throughput tag sequencing) an integrated bioinformatic analysis to identify expression patterns of genes and critical pathways in the female and monoecious lines at three stages of apex development. Assessment of the changes in gene expression between female and monoecious apices yielded sets of up-regulated and down-regulated genes associated with sex expression. Based on these differentially regulated genes (DEGs), some DEGs putatively related to sex expression were selected to confirm by quantitative RT-PCR analysis. Thus, comparison of gene expression patterns at three developmental stages of monoecious and female apices provides important hints for further insight into the molecular mechanisms underlying *Ricinus* sex variation.

## Materials and methods

### Plant material

**Figure 1:**
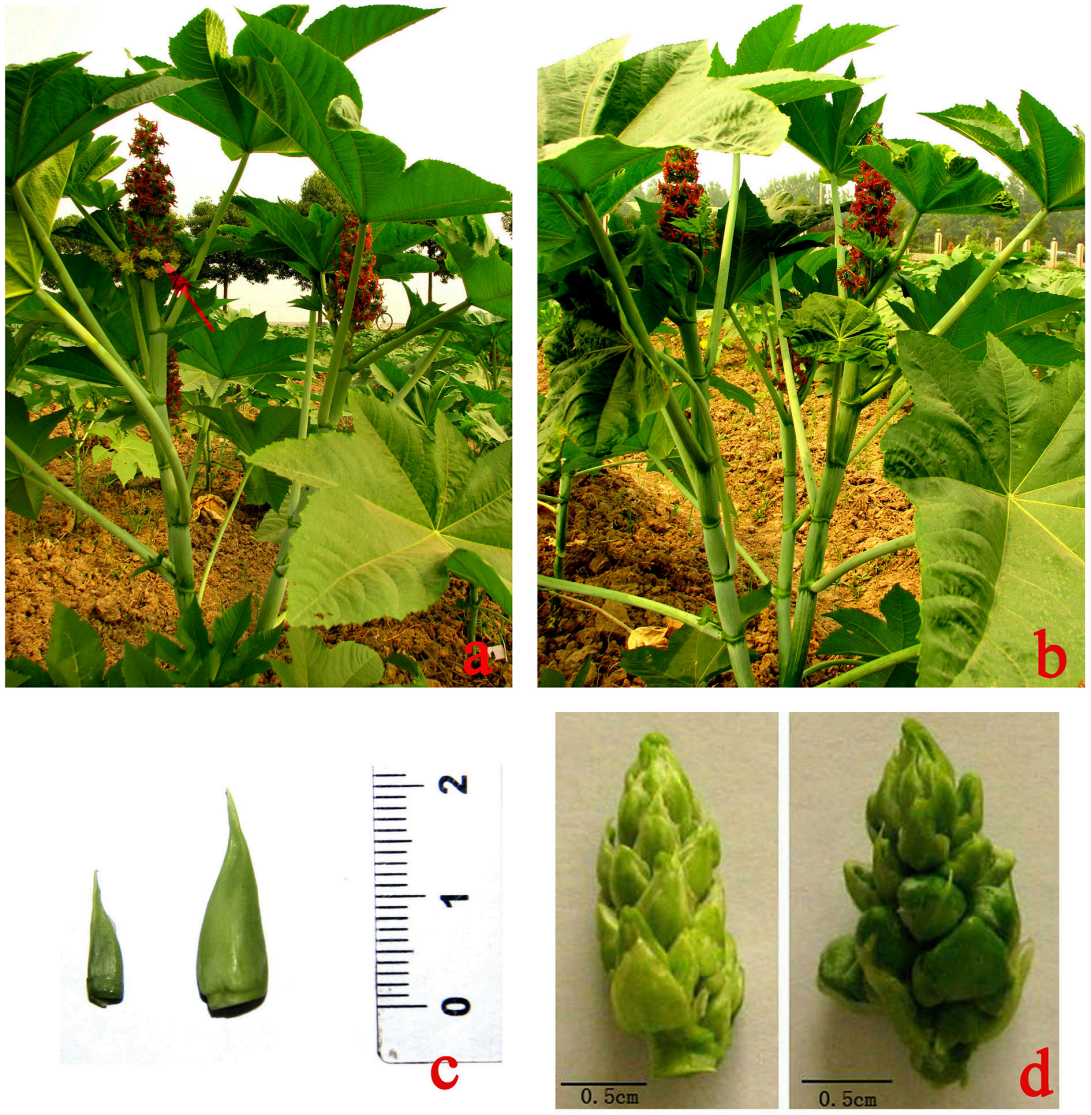
**A *Ricinus* monoecious line (JXBM0705M) and its isogenic female (JXBM0705P); sizes of collected buds and inflorescences for DEG analysis. (A)** monoecious line (JXBM0705M); arrow indicates blooming male flower. **(B)** pistillate line (JXBM0705P); **(C)** collected buds at 3–4-leaf stage (left) and 5–7-leaf stage (right); **(D)** infant female (left) and monoecious (right) inflorescences, 2 cm in length. Scale bars, 0.5 cm.

Seeds of nearly isogenic monoecious (JXBM0705M) and female (JXBM0705P) castor lines were kindly provided by Professor Huang (Castor Oil Research Institute of Jiaxiang, Zibo, Shandong Province). JXBM0705M is a wild monoecious line that bears male and female flowers (Figure 1A), and JXBM0705P, a pistillate line that bears only female flowers (Figure 1B), was developed by consecutively selecting spontaneous mutants of JXBM0705M. Seeds were sown and cultivated in the Yangluo experimental field of the Oil Crops Research Institute of the Chinese Academy of Agricultural Sciences, under standard field conditions, from spring to autumn of 2012. A previous study revealed that apices are leaf buds prior to the 5-leaf stage (i.e., five fully expanded leaves) and change to floral buds during the 6–9-leaf stage (Wang et al., 2012). According to this criterion, and combined with anatomical observation of a series of apical buds of the cultivar, in order to investigate global changes in the transcriptome during the development of buds from leaf apices to racemes, apical buds at the 3–4-leaf stage (ABML1 for monoecism and ABPL1 for female) and the 5–7-leaf stage (ABML2 for monoecism and ABPL2 for female) (Figure 1C), and small racemes 2–3 cm in size (RML for monoecism and RPL for female, Figure 1D), were collected in triplicate to construct 18 DGE libraries. Excised apices and infant racemes were immediately frozen in liquid nitrogen and stored at −80°C until use.

### RNA preparation, library construction, and sequencing

Total RNA was extracted from buds and racemes using the GeneJET Plant RNA Purification Mini Kit (Thermo Fisher Scientific, Waltham, USA). RNA was dissolved in diethylpyrocarbonate (DEPC)-treated water and stored at −70°C. The quality of total RNA was checked on 1% agarose gels, and purity was evaluated by OD260/280 ratio using a NanoDrop ND 1000 spectrophotometer (NanoDrop, Wilmington, DE, USA). Concentration and integrity of RNA were verified using an Agilent 2100 Bioanalyzer. The extracted RNA was prepared for next sequencing and quantitative RT-PCR validation.

A total of 8 μg of total RNA was subjected to oligo (dT) magnetic bead adsorption to purify mRNA. Oligo (dT) was then used as a primer for the synthesis of first- and second-strand cDNA. The 5′-ends of tags could be generated using two types of endonucleases, namely, *Nla*III or *Dpn*II. Bead-bound cDNA was subsequently digested with the restriction enzyme *Nla*III, which recognizes and cleaves CATG sites. Fragments other than 3′-cDNA fragments connected to oligo (dT) beads were washed away, and Illumina adaptor 1 was ligated to the sticky 5′-end of the digested bead-bound cDNA fragments. The junction of the Illumina adaptor 1 and the CATG site constitutes the recognition site for *Mme*I, an endonuclease with separate recognition and cleavage sites. *Mme*I cleaves 17 bp downstream of the CATG site, yielding tags with adaptor 1. After removing 3′ fragments by magnetic bead precipitation, adaptor 2 was ligated to the 3′-ends of tags, yielding tags with different adaptors at both ends to form a tag library. After linear PCR amplification, fragments were purified by PAGE gel electrophoresis. During the quality control steps, the Agilent 2100 Bioanalyzer and ABI Step One Plus Real-Time PCR System were used for quantitation and quality verification of the sample library. Finally, the library was sequenced on an Illumina HiSeq 2000.

### Data analysis and mapping of DGE tags

Millions of raw 49 bp sequences were generated. Image analysis, base calling, generation of raw tags, and tag counting were performed using the Illumina pipeline ('t Hoen et al., 2008). Prior to mapping of tags to the reference database, empty tags (i.e., those with no tag sequence between the adaptors), adaptors, low-quality tags (tags containing one or more unknown nucleotides “N”), and tags with a copy number of 1 were removed from raw sequences to obtain clean tags.

To evaluate the normality of the entire DGE dataset, the distribution of clean tag expression was analyzed. The saturation analysis of the 18 libraries was also performed to estimate whether sequencing depth was sufficient for transcriptome coverage. To identify the gene expression patterns in apical buds and small racemes of two Ricinus lines, all clean tags were annotated by mapping to the castor genome (Chan et al., 2010) using the SOAP2 software (Li et al., 2009a), with a maximum of one nucleotide mismatch allowed. All tags mapped to reference sequences were filtered, and the remaining tags were designated as ambiguous tags. Mapping events on both sense and antisense sequences were included in data processing. For gene expression analysis, in order to identify genes that were differentially expressed between monoecious and female apices at the same developmental stages, as well as genes that exhibited distinctive expression between different developmental stages in the same line, we converted the number of raw clean tags in each library to TPM (number of transcripts per million tags) ('t Hoen et al., 2008; Morrissy et al., 2009) to obtain normalized gene expression levels. Then, nine pairs of DGE profiles of different sample libraries (ABML1 vs. ABPL1, ABML2 vs. ABPL2, RML vs. RPL, ABML1 vs. ABML2, ABML2 vs. RML, ABML1 vs. RML, ABPL1 vs. ABPL2, ABPL2 vs. RPL, and ABPL1 vs. RPL, where a is the control and b is the experimental group in “a vs. b”) were compared to assess the diversity of gene expression. A rigorous algorithm for identifying DEGs (differentially expressed genes) between two samples, false discovery rate (FDR), was applied to determine the threshold *P*-value in multiple tests and analyses. Criteria for DEGs were as follows: FDR, ≤0.001, and |log_2_Ratio|, ≥1 (Audic and Claverie, 1997; Benjamini and Yekutieli, 2001). Following, we performed functional annotation to assign the unambiguously mapped genes identified in all libraries to Gene ontology (GO) terms. Gene annotation was conducted using Blast2GO (Conesa et al., 2005). Gene ontology (GO) was used to determine the possible functions of all DEGs by searching the GO database (http://www.geneontology.org/), and Web Gene Ontology Annotation Plot (WEGO) was also used for GO classification of genes identified in each DGE library (Ye et al., 2006). Moreover, the GO distribution of DEGs in comparisons of each pair of libraries was determined and compared. To further characterize gene function, pathway enrichment analysis of the DGE results was conducted by BLAST search of the KEGG database (http://www.kegg.jp/kegg/). Clustering analysis of differential gene expression patterns was also performed using MultiExperiment Viewer (MeV) (Chu et al., 2008; Howe et al., 2011). A *P*-value of 0.05 was selected as the threshold for a gene set to be considered significantly enriched.

### Quantitative RT-PCR analysis

Quantitative RT-PCR analysis was used to verify the DGE results. The RNA samples used for the qRT-PCR assays were identical to those used for the DGE experiments. Three biological replicates and two technical replicates were performed for each sample. The first-strand cDNA was synthesized from the total RNA (1 ug) from each sample using the PrimeScript RT reagent kit with gDNA Eraser (perfect Real Time) (TaKaRa, RR047A). The primers designed for qPCR analysis was listed in Additional File S1. *Actin* gene (forward primer: 5′-TGCTGACAGAATGAGCAAGG-3′; reverse primer: 5′-AATCCACATCTGCTGGAAGG-3′) was used as an internal control gene for normalization. Quantitative PCR reactions used ABI7500 quantitative PCR, which was performed using SYBR® Premix Ex Taq™ II(Tli RNaseH Plus) (TaKaRa, RR820A) kit according to the manufacturer's instructions. Reaction conditions were 95°C for 5 s in order to activate enzyme reaction. Two step cycles were then used: 95°C for 5 s, then 60°C for 30 s, 40 cycles; solubility curve conditions were 95°C for 15 s, 60°C for 1 min, 95°C for 30 s, 60°C for 15 s. The specificity of the SYBR green PCR signal was further confirmed by melting curve analysis and agarose gel electrophoresis, and the relative expression levels of genes were calculated with the 2^−▵▵Ct^ method.

### Hormone measurements

Contents of several phytohormones including auxin or indole-3-acetic acid (IAA, the major form of auxin in plants), abscisic acid (ABA), jasmonic acid (JA), and gibberellins (GAs) were analyzed to compare and verify the physiological differences between the monoecism and the pistillate. The samples used for the hormone measurement were the same as those used for the DGE experiments (Three development stages of apices from leaf bud to raceme for the monoecism and female, respectively; ABML1, ABML2, and RML for monoecism, ABPL1, ABPL2, and RPL for female.) Three biological replicates were designed for each sample. Quantification of endogenous IAA, ABA, JA, and GAs (GA_1_, GA_3_, GA_4_, and GA_7_) were performed as described previously (Chen et al., 2012). The data of detection was analyzed using Microsoft Excel 2010 and SAS V8 softwares.

## Results

### Analysis of DGE libraries and tag mapping

The 18 DGE libraries were sequenced and generated approximately 6 million raw tags for each library, and more than 96% of the raw tags were clean (Table 1). There produced approximately 5.7 million clean tags per library, of which 148,526–174,340 were distinct. The number of unique distinct clean tag sequences ranged from 134,541 to 159,229 (Table 1). The RPL libraries and ABPL2 libraries contained the two highest numbers of distinct clean tags; the other libraries had similar numbers.

**Table 1:** **Categorization and abundance of tags**.

**Summary**		**ABML1**	**ABPL1**	**ABML2**	**ABPL2**	**RML**	**RPL**
Raw data	Total	5, 919,796 ± 211,429	5, 888,377 ± 122,404	6, 004,837 ± 92,543	6, 000,410 ± 135,373	5, 939,917 ± 123,287	6, 045,455 ± 233,096
	Distinct tags	371,983 ± 4765	374,582 ± 40,008	391,584 ± 12,149	440,819 ± 11,500	369,807 ± 8508	447,852 ± 47,302
Clean tags	Total number	5, 697,507 ± 209,466	5, 661,387 ± 132,174	5, 767,119 ± 88,697	5, 729,206 ± 121,449	5, 722,947 ± 123,544	5, 771,163 ± 203,535
	Distinct tag numbers	150,905 ± 2990	148,526 ± 12,470	155,040 ± 6806	170,590 ± 3920	153,959 ± 4430	174,340 ± 13,817
All tags mapping to genes	Total number	5, 393,163 ± 203,811	5, 397,141 ± 104,629	5, 476,967 ± 99,781	5, 477,295 ± 134,778	5, 490,070 ± 156,338	5, 516,938 ± 151,205
	Total % of clean tags	94.66 ± 0.83	95.35 ± 1.85	94.98 ± 1.72	95.6 ± 0.58	95.92 ± 0.82	95.62 ± 1.79
	Distinct tag numbers	135,847 ± 3278	134,541 ± 8871	139,351 ± 5476	154,604 ± 2420	141,188 ± 2952	159,229 ± 9689
	Distinct tags (% of clean tags)	90.02 ± 0.98	90.69 ± 1.97	89.91 ± 1.71	90.65 ± 1.4	91.72 ± 1.12	91.42 ± 1.89
Unambiguous tag mapping to gene	Total number	168,945 ± 7762	162,305 ± 15,683	163,621 ± 16,927	169,228 ± 5762	172,373 ± 6399	173,617 ± 11,236
	Total % of clean tags	2.96 ± 0.07	2.86 ± 0.21	2.83 ± 0.26	2.96 ± 0.16	3.01 ± 0.09	3.01 ± 0.1
	Distinct tag numbers	7665 ± 306	7323 ± 585	8164 ± 599	8722 ± 416	8223 ± 295	9013 ± 514
	Distinct tags (% of clean tags)	5.08 ± 0.15	4.94 ± 0.31	5.26 ± 0.2	5.11 ± 0.15	5.34 ± 0.04	5.18 ± 0.13
All tag-mapped genes	Number	50,722 ± 577	38,867 ± 1085	45,392 ± 4807	54,090 ± 1159	44,709 ± 1911	41,658 ± 629
	% of ref genes	41.99 ± 0.48	32.17 ± 0.9	37.57 ± 3.98	44.78 ± 0.96	37.01 ± 1.59	34.48 ± 0.52
Unambiguous tag-mapped genes	Number	4867 ± 191	4582 ± 227	5050 ± 333	5215 ± 197	5144 ± 137	5292 ± 144
	% of ref genes	4.03 ± 0.16	3.79 ± 0.19	4.18 ± 0.28	4.32 ± 0.16	4.26 ± 0.12	4.38 ± 0.12
Mapping to genome	Total number	31,478 ± 3790	30,864 ± 8907	33,146 ± 7186	27,758 ± 2213	26,842 ± 2537	30,301 ± 6426
	Total % of clean tags	0.55 ± 0.07	0.54 ± 0.15	0.57 ± 0.12	0.48 ± 0.04	0.47 ± 0.05	0.52 ± 0.1
	Distinct tag numbers	1494 ± 115	1607 ± 173	1879 ± 390	1715 ± 412	1230 ± 113	1548 ± 187
	Distinct tags (% of clean tags)	0.99 ± 0.07	1.08 ± 0.12	1.21 ± 0.2	1 ± 0.22	0.8 ± 0.05	0.89 ± 0.05
Unknown tags	Total number	272,866 ± 43,697	233,382 ± 100,607	257,006 ± 93,700	224,153 ± 28,803	206,034 ± 41,217	223,923 ± 104,814
	Total % of clean tags	4.79 ± 0.76	4.11 ± 1.7	4.45 ± 1.59	3.92 ± 0.55	3.61 ± 0.77	3.85 ± 1.7
	Distinct tag numbers	13,565 ± 1338	12,378 ± 3813	13,810 ± 2853	14,271 ± 2285	11,541 ± 1925	13,563 ± 4350
	Distinct tags (% of clean tags)	8.99 ± 0.91	8.23 ± 1.98	8.89 ± 1.67	8.35 ± 1.18	7.48 ± 1.08	7.69 ± 1.85

**Figure 2:**
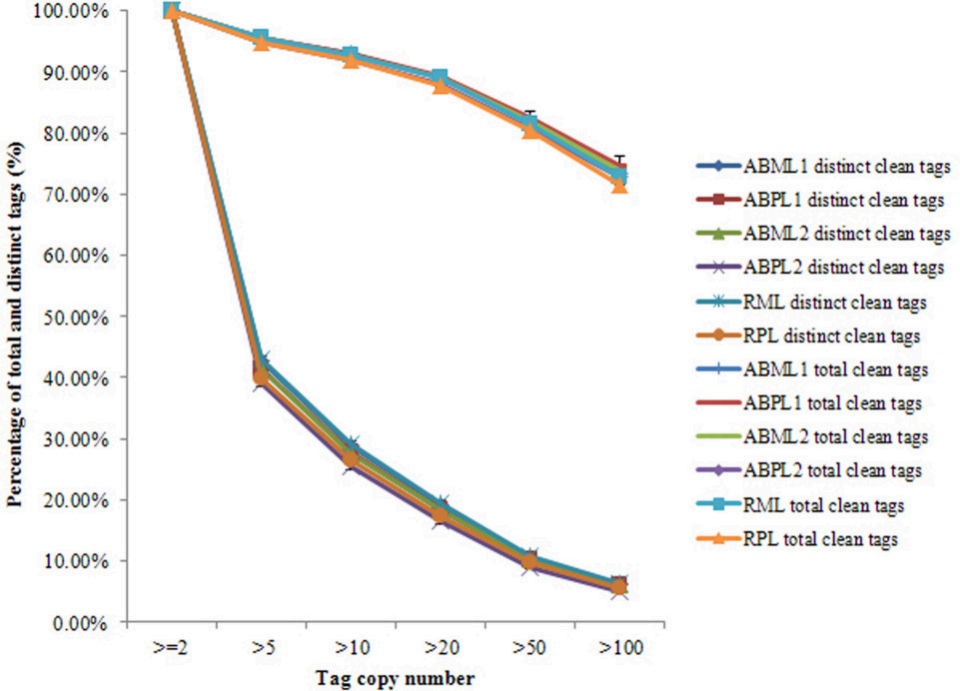
**Distribution of total clean tag and distinct clean tag counts over different tag abundance categories from apical bud and raceme libraries from the monoecious and pistillate lines**.

Our results showed that mRNAs transcribed from major genes were often present at fewer than 10 copies, and only a small proportion of genes were expressed at higher levels (Figure 2; Additional Files S2, S3). The distributions of total and distinct clean tag copy numbers had highly similar properties in libraries from all six sample types, with most tags coming from highly expressed genes (Figure 2). Among the distinct clean tags, more than 56% were present at 2–5 copies, 33.81–36.84% were present at 5–100 copies, and fewer than 6.27% had copy numbers higher than 100. However, more than 70% of total clean tags had counts above 100 in each library, indicating that the overall DGE data among the 18 libraries was normally distributed.

A reference gene database that included 120,799 *R. communis* Unigene sequences was preprocessed for tag mapping. Among the sequences, genes with a CATG site accounted for 90.03% (Additional File S3). Distinct clean tags (4.94–5.34%) were mapped unambiguously in the unigene database, whereas 7.48–8.99% of distinct clean tags were not mapped to the unigene virtual tag database, in libraries from the six sample types (Table 1). Around 95% of total clean tags were mapped onto the *R. communis* genome with a perfect match or 1 bp mismatch to sense or antisense genes, and approximately 90% of distinct clean tags were successfully mapped. Ultimately, tag mapping onto the *R. communis* genome generated 50,722 tag-mapped genes for ABML1, 38,867 for ABPL1, 45,392 for ABML2, 54,090 for ABPL2, 44,709 for RML, and 41,658 for RPL (Table 1; Additional File S4).

In this research, the number of detected genes increased with the amount of sequencing amount until the number of tags reached 3 million or higher (Additional File S5), 20.61–25.73% of distinct clean tags perfectly matched antisense transcripts, and 43.14–53.23% of distinct clean tags perfectly matched sense strand-specific transcripts (Additional Files S3, S4). In total, more than 88% of genes were transcribed from both strands, indicating the importance of RNA-mediated gene regulation in bud transformation from leaf to inflorescence.

### Global gene expression of buds at different stages and analysis of differential gene expression

**Figure 3:**
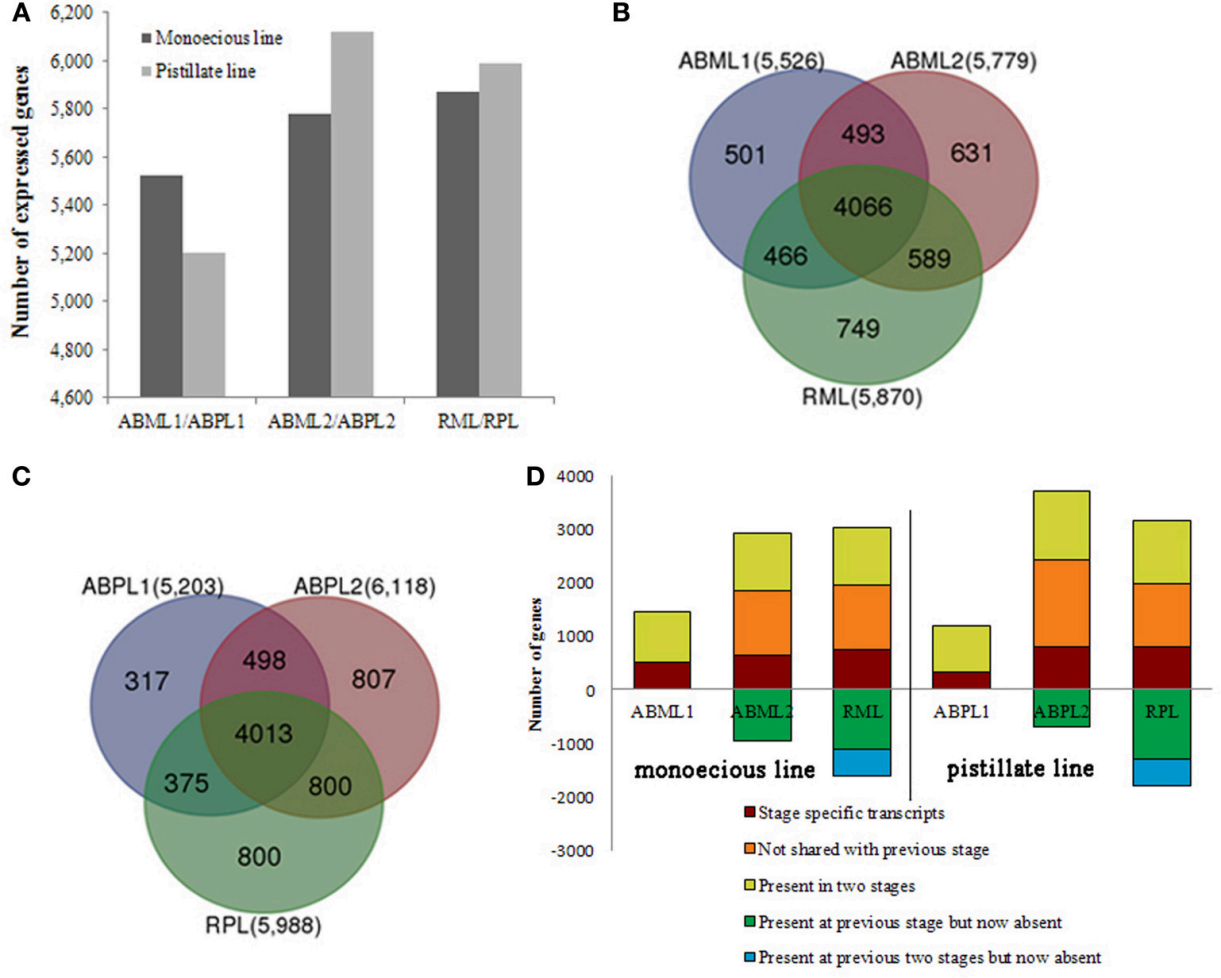
**Transcriptome analysis of apical buds and racemes of the monoecious line (ML) and pistillate line (PL)**. **(A)** Transcriptome sizes of the monoecious and pistillate lines at three developmental stages. **(B,C)** Venn diagram showing the overlaps between three stages (two apical bud stages and raceme stage) of ML and PL. The number in parentheses after each stage designation is the total transcripts detected in that stage(s). **(D)** Analysis of transcriptome changes from apical buds to raceme development of the ML and PL. Transcripts shared by three stages of ML and PL are not shown; numbers above the x-axis represent transcripts present in the indicated stage that are stage-specific (dark red), not shared with the previous stage (orange), or shared with the prior stage but missing in at least one other stage (yellow-green). Numbers below the x-axis represent transcripts present in the prior stage that were not detected in the current stage (green and blue).

In this study, we identified 5526 and 5203 genes in the transcriptomes of monoecious and pistillate apical buds, respectively, at the 3–4-leaf stage; 5779 and 6118 genes at the 5–7-leaf stage; and 5870 and 5988 genes in racemes (Figure 3A).

Our results showed that whether monoecism or female, the transcriptome of their terminal buds during the three developmental stages was dynamic. In total, 7495 and 7610 genes were expressed in monoecious and pistillate apical buds over the three stages, respectively. Of which, 4066 and 4013 were constitutive, 1881 and 1924 were specific to a single stage, and 1548 and 1637 were expressed at two stages, correspondingly for monoecism and female (Figures 3B,C; Additional File S6). The complex changes of gene expression indicated that the transition from leaf apical bud to raceme is an involute process in castor bean. More genes were expressed in the buds closer to raceme formation and incipient racemes than in early apical buds (Figure 3), and more stage-specific transcripts were detected in the 5–7-leaf stage bud and the small raceme than in the early leaf bud (Figure 3D; Additional File S6). These observations suggest that identical molecular pathways are involved in apical bud development from vegetation to reproduction in the monoecism and female, the developmental process may require expression of a larger number of unique genes involved in regulatory processes and related pathways.

**Figure 4:**
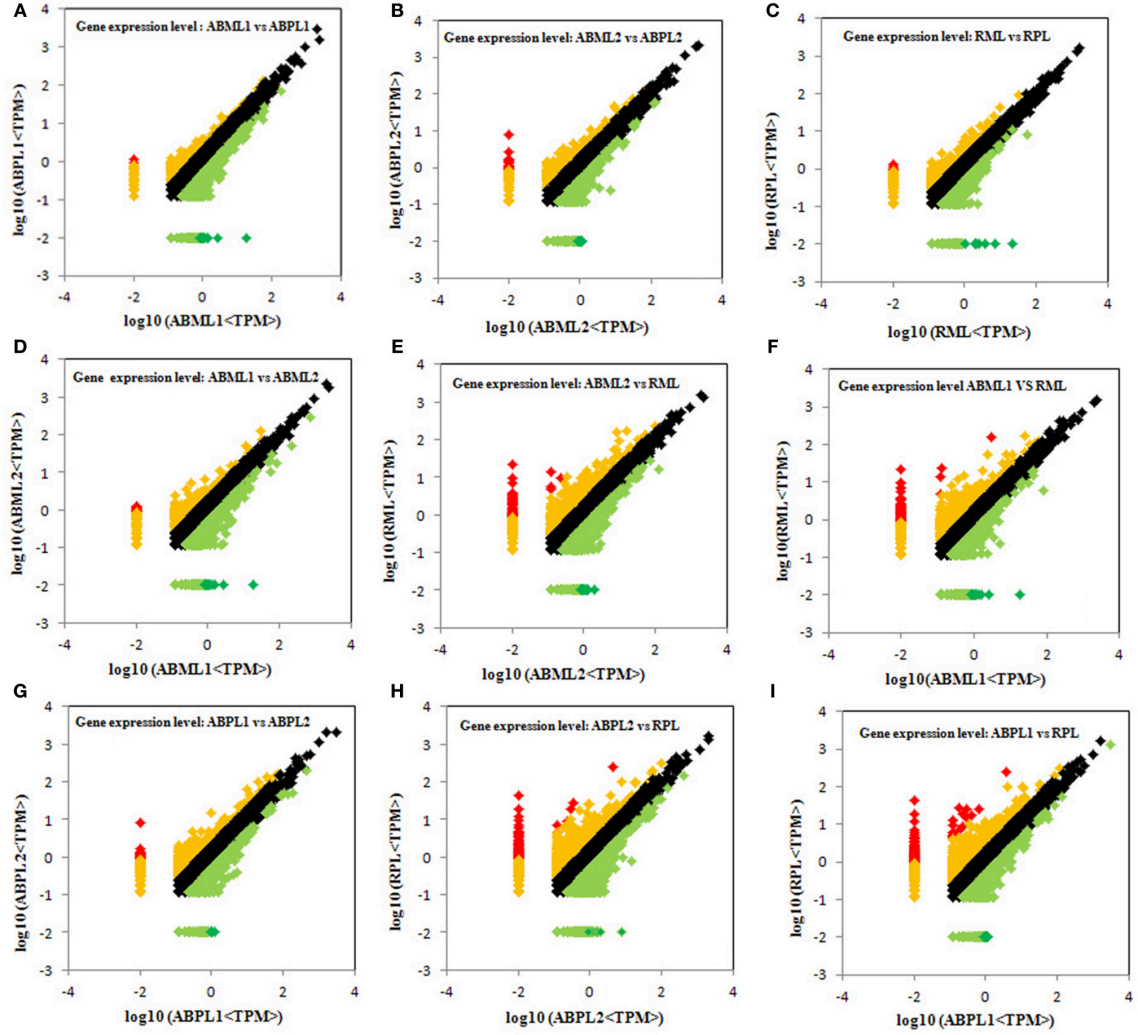
**Comparison of gene expression levels across all libraries**. All genes mapped to the reference sequence were examined for differential expression across the libraries. ABML1 and ABPL1: apical bud of 3–4-leaf stage of monoecious and female lines, respectively; ABML2 and ABPL2: apices of 5–7-leaf stage of monoecious and pistillate lines; RML and RPL: small raceme (2–3 cm long) of monoecious and female lines. Red prism represented significantly up-regulated genes (log2Ratio, ≥1; probability, ≥0.8), yellow represented up-regulated genes (log2Ratio, ≥1; probability, <0.8), black showed not DEGs (log2Ratio, ≥0 and <1), light green indicated down-regulated genes (log2Ratio, ≤ −1; probability, <0.8) and green prism demonstrated significantly down-regulated genes (log2Ratio, ≤ −1; probability, ≥0.8).

Comparison of the monoecious line with the female line revealed that 1386 DEGs were up-regulated and 1946 DEGs were down-regulated at the 3–4-leaf stage, but the majority of genes were up-regulated during the subsequent two stages: 1952 up-regulated and 1408 down-regulated genes at the 5–7 leaf stage, and 1667 up-regulated and 1451 down-regulated genes at the initial raceme stage (Table 2; Figures 4A–C; Additional File S7). The number of up-regulated DEGs in the female was greater than the number of down-regulated DEGs during the developmental transition from leaf bud to infant raceme, which might indicate that females require a number of genes involved in silencing of male flower expression. Similarly, it was showed that up-regulated genes outnumbered down-regulated genes in these comparisons between different development stages, except for the RPL vs. ABPL2 (Table 2; Figures 4D–I; Additional File S7), which also revealed that the expression of many genes tended to increase; a few genes were activated, whereas others were suppressed, during bud development from vegetation to reproduction.

**Table 2:** **Gene expression levels across different sample libraries**.

	**ABML1/ABML2**	**ABML2/RML**	**ABML1/RML**	**ABPL1/ABPL2**	**ABPL2/RPL**	**ABPL1/RPL**	**ABML1/ABPL1**	**ABML2/ABPL2**	**RML/RPL**
Significantly up-regulated	17	48	68	32	75	103	2	15	6
Up-regulated	1873	1991	2083	2613	2055	2535	1384	1937	1661
Not DEGs	3372	3184	3207	3043	2940	2863	3089	3664	3855
Down-regulated	1471	1757	1494	1118	2190	1293	1929	1401	1443
Significantly down-regulated	13	14	12	4	33	9	17	7	8
Total expression genes	6746	6994	6864	6810	7293	6803	6421	7024	6973

Significantly up-regulated: log_2_Ratio, ≥1, and probability, ≥0.8; Up-regulated: log_2_Ratio, ≥1, and probability, <0.8; Not DEGs: genes not differentially expressed (0 ≤ |log_2_Ratio|<1); Down-regulated: log_2_Ratio, ≤ −1, and probability, <0.8; Significantly down-regulated: log_2_Ratio, ≤ −1, and probability, ≥0.8.

### Functional annotation and DEG clustering analysis

Go analysis revealed that these well-annotated sequences belonged to three main categories (cellular component, molecular function, and biological process) and mainly distributed into 43 categories, including the most predominant pathways such as “Intracellular membrane-bounded organelle,” “Adenyl ribonucleotide binding,” “Transition metal ion binding,” “Intrinsic to membrane,” “Cellular protein modification process,” “Plastid,” “Membrane and cell part,” “Binding and protein binding,” “Metabolic process,” and “Transcription, DNA-dependent.” The distributions among the pairwise comparisons were similar, except for small differences in the numbers of genes in each category and the total numbers of main category (Additional Files S8, S9). In addition, all of the annotated genes were mapped to terms in the KEGG database to search for significantly enriched genes involved in spliceosome, cell cycle, homologous recombination, or signaling pathways (Additional File S10).

**Figure 5:**
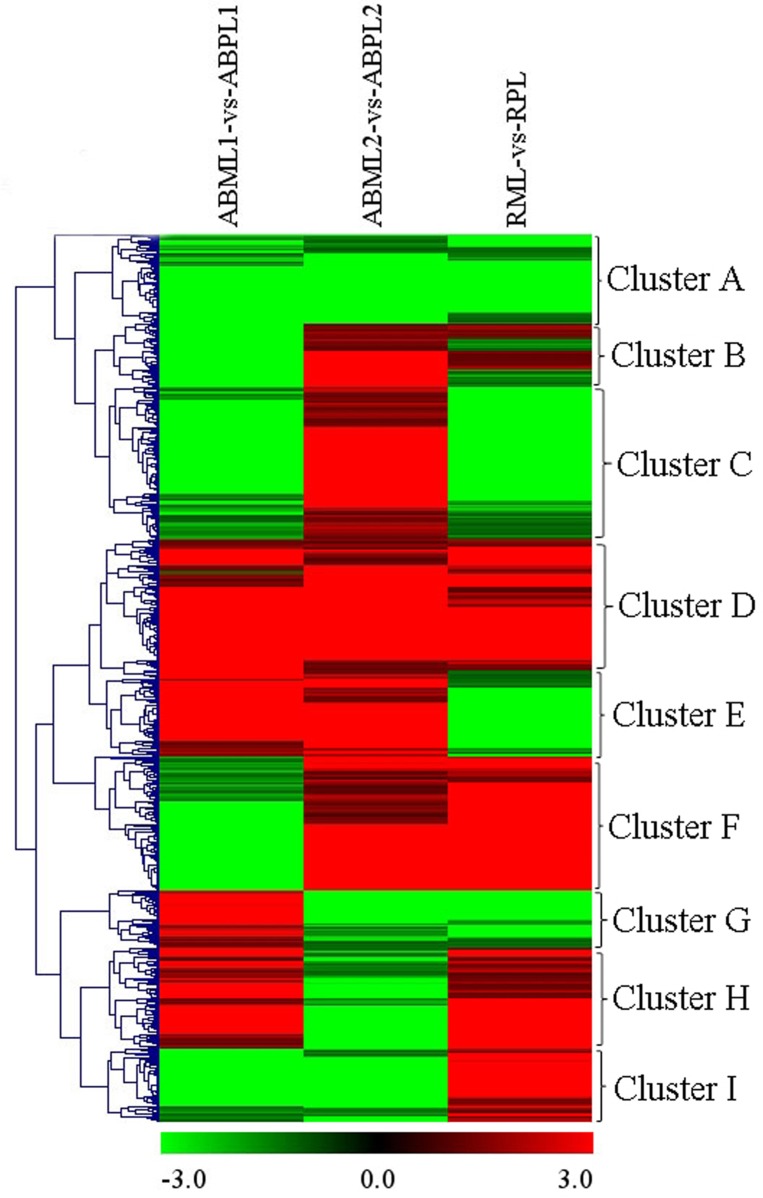
**Hierarchical clustering (HCE) analysis of differentially expressed transcripts between monoecious and pistillate lines at three development periods (ABML1 vs. ABPL1, ABML2 vs. ABPL2, and RML vs. RPL**. “a” was the control and “b” was experimental group in “a vs. b”). The clusters from A to I indicate the nine major clusters resulting from HCE analysis. Each line refers to data from one gene. The color bar represents the log_10_RPKM and ranges from green (low expression) to red (high expression).

In general, the 7687 DEGs in all comparisons were clustered as the union of DEGs. A total of 617 transcripts occurring simultaneously in the three comparisons of three development stages between the monoecism and female were identified, and the comparison of the ratio values of these genes were used for clustering as the intersections of DEGs (Figure 5). Among the nine major clusters, 62 genes grouped into Cluster A were down-regulated in the three comparisons of developmental stages between the monoecism and the female, whereas the 97 genes in Cluster D were up-regulated in the three comparisons. GO analysis of these clustered genes indicated that the known genes were mainly involved in membrane or vesicle structures of intracellular or cytoplasmic components, activity and binding functions, response to light or hormone stimulus, transport, signal transduction, metabolic processes, and developmental processes involved in differentiation and reproduction (Additional File S11).

**Figure 6:**
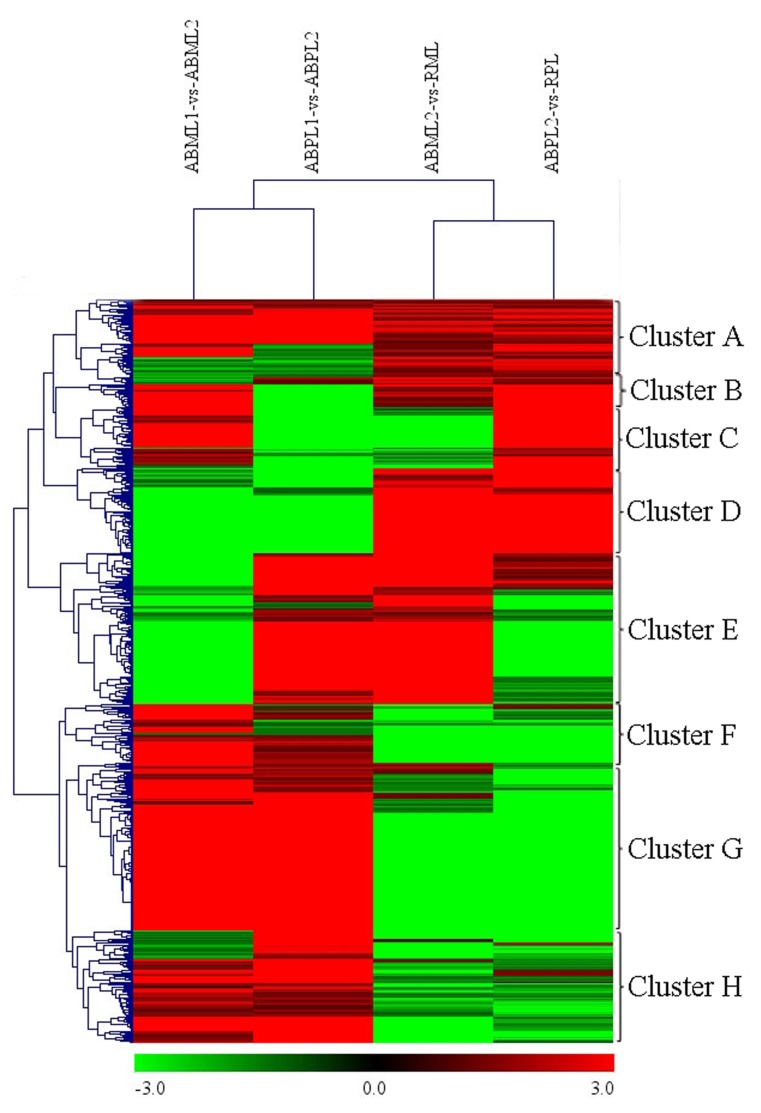
**Hierarchical cluster analysis of DEGs between different developmental stages for the monoecious and female lines (ABML1 vs. ABML2, ABML2 vs. RML, ABPL1 vs. ABPL2, and ABPL2 vs. RPL**. “a” was the control and “b” was experimental group in “a vs. b”). Each line refers to data from one gene. The color bar represents the log_10_RPKM and ranges from green (low expression) to red (high expression). The clusters from A to H indicate the eight major clusters resulting from HCE analysis.

A heat map was generated of DEGs between different growth stages for both the monoecious and female buds (Figure 6). There were 621 DEGs in the four comparisons that clustered as the intersections of DEGs (Additional File S12). As shown in Figure 6, the DEGs in “ABML1 vs. ABML2” and “ABPL1 vs. ABPL2” were closely correlated to the DEGs in “ABML2 vs. RML” and “ABPL2 vs. RPL,” respectively, indicating that gene expression differences between monoecious and female lines were not obvious during the same development processes. However, this analysis revealed a greater number of DEGs between different development processes, with a relatively distant relationship, which suggests that transformation of apical bud from leaf to flower is a complex process.

### Genes associated with sex expression and reproduction in castor bean

Based on prior knowledge of the putative involvement in sex determination and the expression levels and functions of DEGs between the monoecious and female buds in three development stages, several subgroups were assumed to be putatively related to sex expression and reproduction (Table 3). We identified seven genes involved in response to hormone stimulus, whose expression changed significantly in mutant females over the three developmental stages (Table 3). These genes included those that encode dynamin-2A, auxin response factor, PCI domain-containing protein, ATP-binding protein, spermidine synthase, Xaa-Pro amino peptidase, and a conserved hypothetical protein. These genes are associated with tissue or organ developmental processes, and several of them take part in signal transduction, auxin transport, phytohormone biosynthesis, and metabolism (such as polyamine and abscisic acid).

**Table 3:** **Some selected differentially expressed genes detected by digital expression profiling in apical bud of castor bean**.

**Functional group**	**Gene ID**	**Gene annotation**	**log**_**2**_**ratio**		
			**ABML1/ABPL1**	**ABML2/ABPL2**	**RML/RPL**
**RESPONSE TO HORMONE STIMULUS**					
	gi|255573875|ref|XM_002527811.1|	Dynamin-2A	−8.05	−3.54	−8.54
	gi|255538885|ref|XM_002510462.1|	Auxin response factor (transcription DNA-dependent)	−3.58	1.00	−3.66
	gi|255546086|ref|XM_002514057.1|	PCI domain-containing protein	−3.66	3.58	4.84
	gi|255543197|ref|XM_002512616.1|	ATP-binding protein	1.15	−6.23	4.84
	gi|255550142|ref|XM_002516076.1|	Spermidine synthase (auxin transport, polyamine biosynthetic process)	−5.71	1.36	−1.54
	gi|255568254|ref|XM_002525056.1|	Xaa-pro amino peptidase (auxin transport)	−1.77	1.50	−1.78
	gi|255560413|ref|XM_002521176.1|	conserved hypothetical protein (ABA and carotenoid metabolic process)	−4.56	4.56	3.58
**TRANSCRIPTION, DNA-DEPENDENT**					
	gi|255558565|ref|XM_002520262.1|	MADS box protein	−2.52	−4.90	−2.80
	gi|255583281|ref|XM_002532359.1|	DNA-binding protein	3.58	3.66	3.58
	gi|255572532|ref|XM_002527155.1|	Axial regulator YABBY5	−1.18	8.07	5.69
	gi|255538455|ref|XM_002510247.1|	DNA-binding protein	−2.08	1.31	4.14
	gi|255558973|ref|XM_002520464.1|	transcription initiation factor	3.58	−3.58	3.58
	gi|255565967|ref|XM_002523926.1|	RNA polymerase sigma factor rpoD1	−5.14	−4.54	4.14
	gi|255558989|ref|XM_002520472.1|	protein with unknown function	−1.15	−1.80	3.58
	gi|255538885|ref|XM_002510462.1|	Auxin response factor	−3.58	1.00	−3.66
**SIGNAL TRANSDUCTION**					
	gi|255555566|ref|XM_002518774.1|	gcn4-complementing protein	−5.16	−1.07	−4.56
	gi|255550049|ref|XM_002516030.1|	protein with unknown function	−4.92	3.62	−1.57
	gi|255585732|ref|XM_002533502.1|	Histidine-containing phosphotransfer protein	−5.39	4.87	−3.62
	gi|255584316|ref|XM_002532848.1|	Cyclic nucleotide-gated ion channel	−3.58	3.66	−4.14
**SEX DIFFERENTIATION**					
	gi|255557726|ref|XM_002519847.1|	arginine/serine-rich splicing factor	−4.56	3.66	3.58
**POLLINATION**					
	gi|255560126|ref|XM_002521035.1|	acid phosphatase	−2.42	1.27	1.90
**PROGRAMMED CELL DEATH**					
	gi|255539309|ref|XM_002510674.1|	*Ricinus communis* cysteine protease	−4.22	3.66	−4.54
**DEVELOPMENTAL PROCESS INVOLVED IN REPRODUCTION**					
	gi|255577146|ref|XM_002529411.1|	1,4-alpha-glucan branching enzyme	−1.02	4.20	−3.62
	gi|255588742|ref|XM_002534658.1|	DNA replication helicase dna2	−1.45	5.71	−1.96
	gi|255568769|ref|XM_002525310.1|	sentrin/sumo-specific protease	−1.66	2.32	1.29
	gi|255578470|ref|XM_002530054.1|	U4/U6 small nuclear ribonucleoprotein Prp4	−6.24	4.20	4.22
	gi|255544821|ref|XM_002513426.1|	sorting and assembly machinery (sam50) protein	−3.66	4.20	4.14
**HISTONE DEMETHYLATION/METHYLATION**					
	gi|255565532|ref|XM_002523711.1|	eukaryotic translation initiation factor 2c	−3.58	1.29	1.67
	gi|255575536|ref|XM_002528623.1|	set domain protein	2.78	−4.20	−4.14
	gi|255553976|ref|XM_002517983.1|	DNA (cytosine-5)-methyltransferase	−4.89	3.58	−3.62
	gi|255558643|ref|XM_002520301.1|	s-adenosyl-methyltransferase mraw	1.23	3.66	5.33

In this study, we detected eight transcription factors that exhibited significantly different expression patterns between the pistillate and monoecious lines during the three developmental stages. These transcriptional regulators were identified as DNA-dependent transcription factors, including MADS box proteins, DNA-binding proteins, the Axial regulator YABBY5, transcription initiation factors, auxin response factor, and the RNA polymerase sigma factor rpoDI. Auxin response factor is respond to hormone stimulus and participates in hormone-mediated signaling pathways, also plays a role in tissue and organ developmental processes. Furthermore, one subgroup is composed of four genes related to signal transduction, including Gcn4-complementing protein, histidine-containing phosphotransfer protein, an unknown protein, and cyclic nucleotide-gated ion channel, which exhibited consistent expression changes: down-regulation at the early leaf-bud stage, up-regulation at the 5–7-leaf stage, and down-regulation at the young raceme stage. Another two genes listed above (PCI domain-containing protein and ATP-binding protein) play roles in signal transduction in addition to response to hormone stimulus.

One gene (*arginine/serine-rich splicing factor*) associated with sex differentiation and another gene (*acid phosphatase*) related to pollination were identified as differentially expressed between monoecious and pistillate apical bud development. In female apical buds, both genes were distinctly down-regulated at the early leaf-bud stage, but up-regulated at the 5–7-leaf and initial raceme stages. In addition, expression of a PCD (programmed cell death)-related gene that encodes cysteine proteinase was altered in pistillate apices, with down-regulation at the early leaf-bud and initial raceme stages and up-regulation during the period of transition from leaf bud to floral bud. Several genes involved in reproduction exhibited the same expression patterns in pistillate terminal bud: sentrin/sumo-specific protease, the U4/U6 small nuclear ribonucleoprotein Prp4, sorting and the assembly machinery (sam50) protein were down-regulated at the early leaf stage, but up-regulated at the 5–7-leaf and small raceme stages, (Table 3); notable exceptions were 1,4-alpha-glucan branching enzyme and DNA replication helicase (*dna2*). Histone modification and DNA methylation or demethylation, play an important role in the regulation of gene expression or silencing during developmental processes. We identified four genes involved in histone methylation and demethylation (Table 3), including eukaryotic translation initiation factor 2c, set domain protein, DNA (cytosine-5)-methyltransferase, and s-adenosyl-methyltransferase (*mraw*). These genes were also significantly differentially expressed between the monoecious and female apical bud development.

### DEGs were confirmed by quantitative RT-PCR

**Figure 7:**
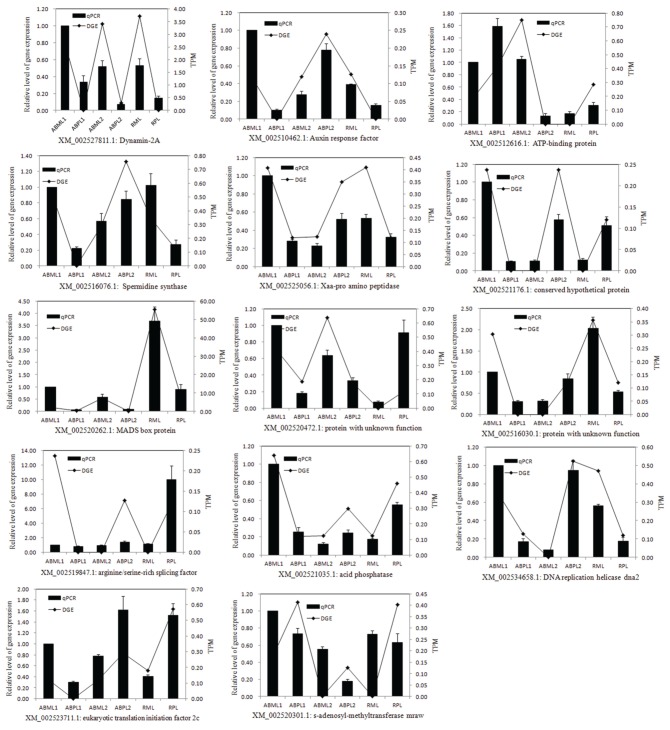
**Quantitative RT-PCR validation of differential expressed genes**. TPM, Transcription per million mapped reads. ABML1 and ABPL1, apical buds at 3–4-leaf stage of monoecious and pistillate lines; ABML2 and ABPL2, apical buds at 5–7-leaf stage of monoecious and pistillate lines; RML and RPL, infant raceme stage of monoecious and female lines. Relative expression levels were calculated using Actin as an internal control. Error bars indicate SE (*n* = 3).

To confirm the differentially expressed genes in the monoecious and female apical buds, 14 genes were selected for quantitative RT-PCR analysis at the three apical bud developmental stages. Representative genes selected for qPCR validation were those involved in response to hormone stimulus, transcription factor, signal transduction, sex differentiation, pollination, reproduction and histone demethylation/methylation because those phenomena were putative to associate with plant sex determination(Guo et al., 2010; Wu et al., 2010). The expression of the 13 genes (Dynamin-2A, Auxin response factors, ATP-binding protein, Spermidine synthase, auxin transport, conserved hypothetical protein, MADS box protein, two unknown proteins, arginine/serine-rich splicing factor, acid phosphatase, DNA replication helicase dna2, and eukaryotic translation initiation factor 2c) indicated by qRT-PCR was basically congruent with the DGE analysis patterns (Figure 7). Only one gene (s-adenosyl-methyltransferase mraw) did not show consistent expression between the qRT-PCR and DGE data sets.

### Differential hormone levels in the apices and racemes between the monoecism and female

**Figure 8:**
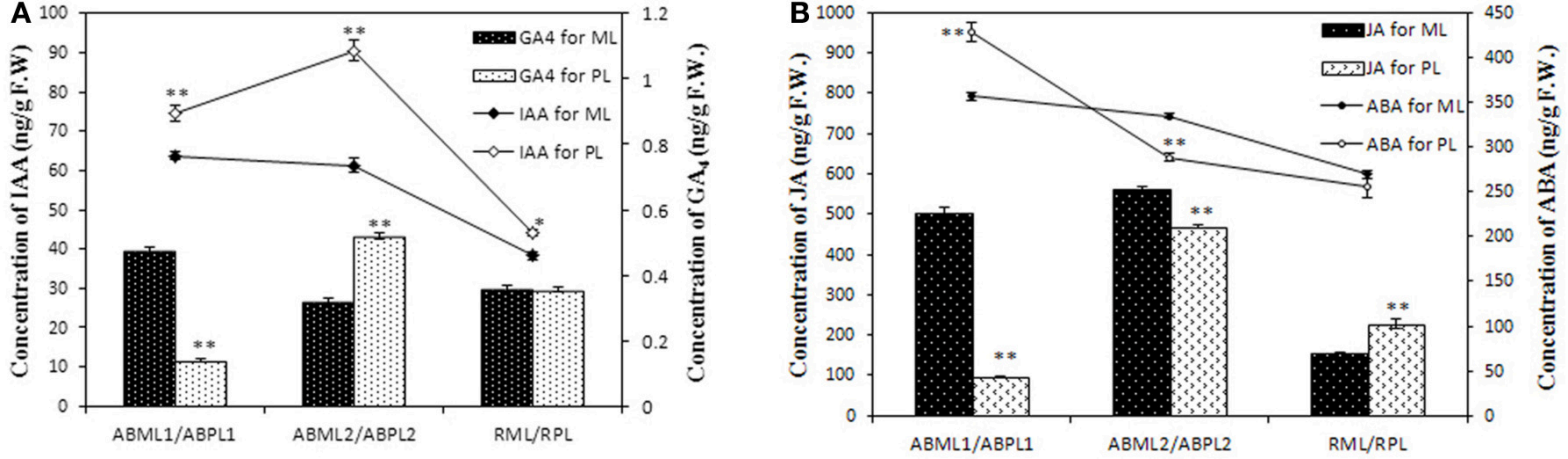
**Levels of IAA, GAs, ABA, and JA hormones in the apices and racemes of the monoecious line and pistillate line**. ML, monoecious line; PL, pistillate line; ABML1 and ABPL1, apical buds at 3–4-leaf stage of monoecious and pistillate lines; ABML2 and ABPL2, apical buds at 5–7-leaf stage of monoecious and pistillate lines; RML and RPL, infant raceme stage of monoecious and female lines; F.W., fresh weight. **(A)** IAA and GA_4_contents of apices and racemes for ML and PL; **(B)** JA and ABA levels in apices and racemes of ML and PL. Error bars indicate SE (*n* = 3); Asterisks indicate *p* < 0.05 (^*^) and *p* < 0.01 (^**^) between PL and ML in Duncan's test analysis.

DGE analysis and qRT-PCR verification showed that some genes which response to hormone stimulus were expressed differentially in apices and racemes between the monoecism and female, and possibly associated with castor sex expression. To validate whether the levels of phytohormone in the apices and inflorescences of the monoecious line and the pistillate line vary or not, the IAA, ABA, JA, and GAs contents of the two lines at three developments were quantitatively measured. The result showed that the auxin in the tow castor lines indicated a similar variation pattern in that IAA was equivalent or increased from the 3–4-leaf stage to the 5–7-leaf stage, and later significantly reduced (*P* < 0.01) (Figure 8A; Additional File S13). It was worthwhile to note that the auxin level in pistillate line was remarkably higher than that in the monoecism at each of the three development stages. Four kinds of GAs were performed in the measurement, but only GA_4_ were detected with a relatively low level, and other three GA were not detected. The GA_4_ level in the apices except little racemes also showed significant differences between the monoecism and female (Figure 8A; Additional File S13). The level of GA_4_ in the female was significantly lower (*P* < 0.01) than that in the monoecism at the stage of early leaf bud, but markedly higher (*P* < 0.01) at the 5–7-leaf stage and similar at the stage of young inflorescence.

The ABA levels in the monoecism and female revealed an identical change pattern from early leaf apices to infant racemes, with a notable consecutive reduction (Figure 8B; Additional File S13). The ABA level in female was significantly higher (*P* < 0.01) than that in monoecism at early leaf apices, but notable lower (*P* < 0.01) at following 5–7-leaf stage, and comparative level at little raceme stage. In addition, the variation in endogenous JA levels showed a similar pattern as IAA in the two castor lines during the three development stages (Figure 8B; Additional File S13). However, opposite to IAA, the JA level in the pistillate line was extremely significant lower (*P* < 0.01) than that in the monoecism at the two apical development stages, and significantly higher (*P* < 0.01) than that in monoecism at the stage of little raceme.

## Discussion

Sex expression in plants is controlled by genetic factors and non-genetic fluctuations (Shifriss, 1956; Ainsworth, 1999), and phytohormones play important roles in sex determination in some species (Yin and Quinn, 1995; Ainsworth, 1999; Tanurdzic and Banks, 2004). In cucumber, ethylene, a true sex hormone, was proven to be a “female” hormone that exerts a strong feminizing effect (Dellaporta and Calderon-Urrea, 1993); Auxin also promotes cucumber femaleness, potentially playing an indirect role in sex determination by promoting the action of ethylene (Yamasaki et al., 2000). The level of gibberellic acid (GA), which acts downstream of ethylene or through a parallel pathway (Ainsworth, 1999), is correlated with male tendency. Sex expression in castor bean is also regulated by plant hormones (Shifriss, 1961; Tan et al., 2011), but in contrast to cucumber, ethylene and ethylene-like substances (NIA 10637) result in masculinization and can transform female flowers into male ones in monoecious plants (Philipos and Narayanaswamy, 1976); moreover, GA causes feminization in castor, and spraying monoecious inbreds with GA can markedly increase female tendency (Shifriss, 1961). In addition, previous work showed that the activity of auxin-like substances causes feminization of castor bean, a process influenced by kinetin and morphactin (Kumar and Rao, 1981). Morphactin causes maleness in *R. communis* (Rkey, 1978; Varkey and Nigam, 1982), whereas daminozide promotes femaleness with reduction in the position of bearing the first female flower and the ratio of male and female flowers (Chauhan et al., 1987). To date, however, the key sex hormone in castor bean remains unknown. In this study, we identified several genes involved in the response to hormone stimulus (Table 3; Figure 7), including auxin transport and auxin response factor genes and other genes involved in plant hormone-mediated biosynthetic and metabolic process, such as polyamine biosynthesis and abscisic acid and carotenoid metabolism. In the pistillate line, three genes (*auxin response factor, spermidine synthase*, and *Xaa-Pro amino peptidase*) related to auxin response and transport, were down-regulated in early leaf apices and young racemes, but up-regulated during the transformation from leaf apices to floral buds. In addition, the result of hormone measurement detected that auxin level in the female was higher than that in the monoecism at each of the three development stages and extremely significant higher at the 5–7-leaf stage. The levels of JA, ABA, and GA in the apices also displayed significant differences between the pistillate line and the monoecious line, except the GA and ABA level in the infant racemes (Figure 8). Our results suggested that DEGs involved in the pathways of plant hormone response and hormone-mediated biosynthetic and metabolic processes, may participate in or affect the regulation of sex expression and reproduction by changing the hormone level and transport, which will be elucidated in further research. Our result was consistent with previous reports (Heslop-Harrison, 1957; Rkey, 1978). Those authors showed that variation in sex expression in plants is associated with variations in the hormone level (Heslop-Harrison, 1957), and that auxins promote femaleness (Wittwer and Hillyer, 1954). Interference in auxin transport, or quick degradation of the hormone, can decrease the auxin level, resulting in altered sex expression (Thomson and Leopold, 1974; Gaganias and Berg, 1977). The fact that lateral branches of castor bean plants produce only male flowers after decapitation supports the view that inhibition of auxin transport can result in maleness (Rao, 1969). Furthermore, our previous research (Wang et al., 2012) demonstrated that IAA and ABA levels in the inflorescence and flower vary remarkably between the pistillate and the monoecism, also suggesting that some phytohormones such as IAA etc. and related genes are important for sex determination in *R. communis*. In plants, phytohormones modulate various growth and developmental events through signal transduction pathways (Bleecker and Kende, 2000; Davies, 2004). Notably, in this study, three genes involved in signal transduction exhibited expression patterns similar to those of genes involved in auxin response and transport (i.e., down-regulated in early leaf apices and small racemes, but up-regulated during the transformation stage from leaf to floral bud) in the pistillate line. However, other genes involved in signal transduction were down-regulated throughout all three stages. Subsequent investigations of these genes and crosstalk between other hormones and auxin may reveal their specific roles in sex expression or sex determination.

Castor is a monoecious species, with pistillate flowers borne on short pedicels along the upper portion of the raceme and staminate flowers borne similarly on the lower portion (Brigham, 1967). Castor flowers are unisexual and lack petals. Male and female flowers differ radically in general morphology and size: male flowers are devoid of rudiments of organs of the opposite sex, whereas pistillate flowers occasionally exhibit vestiges of stamen (Wu et al., 1998). The occasional appearance of rudimentary androecium in *Ricinus* female flowers suggests that like unisexual flowers of other species (Atsmon and Galun, 1960; Cheng et al., 1983; Bracale et al., 1991; Malepszy and Niemirowicz-Szczytt, 1991; Veit et al., 1993), castor flowers (especially pistillate flowers) pass through a “bisexual stage” during which development of all floral organs is initiated. Castor sex determination probably involves PCD of opposite sex organs in the flower and inflorescence, similar to what is observed in several other plant species such as maize, cucumber, and campion (Ye et al., 1991; Dellaporta and Calderon-Urrea, 1993; Kater et al., 2001); these plants follow a sex determination pathway involving arrest of preformed sexual organs in bisexual primordia in which some PCD-related genes participate (DeLong et al., 1993). In this study, we detected a PCD-related gene (cysteine protease) that was significantly up-regulated in female apices at the 5–7-leaf stage, but obviously down-regulated at the two other stages (early leaf apices and initial inflorescence). This result was consistent with recent findings that PCD-related genes, including cysteine protease, are more highly expressed at the peak of anther abortion in CMS and GMS lines of cotton (Lorrain et al., 2003; Wei et al., 2013). It is possible that the cysteine protease might play a role in *Ricinus* apical development, and thus be associated directly or indirectly with sex expression. Our study identified hundreds of genes potentially involved in sex determination, including one (*arginine/serine-rich splicing factor*) associated with sex differentiation; this gene was up-regulated during apical transformation and initial raceme formation in female apical buds.

Transcription factors such as the maize DELLA protein D8 and the melon zinc finger protein CmWIP1 have been functionally associated with the sex determination process (Peng et al., 1999; Martin et al., 2009). Moreover, several transcription factors were identified as differentially expressed during sex determination in cucumber (Guo et al., 2010; Wu et al., 2010), including zinc finger transcription factors, Aux/IAA transcription factor, auxin-induced protein, BRI1, BRI1-associated receptor kinase, MYC transcription factor, BEL1-like homeodomain protein, bHLH proteins, WRKY DNA-binding protein, and NAC domain protein, *IF-2*, and so on. Consistent with the results in cucumber, we identified eight DNA-dependent transcription genes in castor bean that were significantly differentially expressed between the three apical developmental stages in the pistillate line. Of these, auxin response factor, MADS box protein, *IF-2*, and two DNA-binding proteins were also confirmed to be differentially expressed during sex determination. These transcription factors detected in this study were mainly binding proteins, response factors, initiation and regulation factors, and even MADS box proteins; the relationship between these transcription factors and plant sex determination remains to be determined, and should be investigated in future work. In addition, we also identified a few genes involved in histone demethylation/methylation and reproductive processes. DNA methylation and histone modification play essential roles in genome management and control gene expression or silence (Cheng and Blumenthal, 2008). DNA methyltransferase, the main enzyme involved in DNA methylation, was detected in female apices; it was obviously up-regulated at the stage of transformation from leaf to floral apices, and down-regulated at the other two developmental stages, relative to monoecious apices (Table 3). DNA methyltransferase is not only associated with DNA methylation, but is also associated with many important biological activities, including cell proliferation and senescence (Berger, 2007). The increase in methyltransferase gene expression during the transitional stage may change the level of DNA methylation, leading to the subsequent repression of male flower gene expression and the appearance of only female flowers in female racemes. Genes associated with the reproduction process, identified as DEGs here and in future research, will contribute to elucidation of the molecular mechanisms underlying *Ricinus* sex determination and sex patterns.

## Data deposition

The Illumina pair-end reads of *Ricinus communis* L. obtained in the study are available at NCBI SRP064760.

## Author contributions

YX conceived and designed the study, amended the manuscript. TM participated in its design and performed the experiments, analyzed the data and drafted the manuscript. XJ and WL prepared the samples, RNA extraction, cDNA library construction and helped with the sequencing. FC collected and prepared the samples. HJ provided the castor seeds and took part in field cultivation and bud collections. All authors read and approved the final manuscript.

## Funding

This work was jointly supported by the National Natural Science Foundation of China (Grant No. 31271765 and No. 31000726), the National Department (Agriculture) Public Benefit Scientific Research Foundation (Grant No. 201003057), Ministry of Science and Technology, Ministry of Finance and the National Science and Technology Infrastructure Platform (NICGR2015-014).

### Conflict of interest statement

The authors declare that the research was conducted in the absence of any commercial or financial relationships that could be construed as a potential conflict of interest.

## References

[B1] Ainsworth C. C. (1999). Sex Determination in Plants. Oxford: BIOS Scientific Publishers.

[B2] Amaral J. G. C. (2003). Genetic *Variability* for *Agronomic Characteristics* between self *Pollinated Lines* of *Castor* (Ricinus communis L.) cv. AL *Guarany*. Ph.D. dissertation (Agronomy), College of Agronomic Sciences, São Paulo State University, Botucatu.

[B3] Anjani K. (2012). Castor genetic resources: a primary gene pool for exploitation. Ind. Crop Prod. 35, 1–14. 10.1016/j.indcrop.2011.06.011

[B4] Atsmon D. Galun E. (1960). A morphogenetic study of staminate, pistillate and hermaphrodite flowers in *Cucumis sativus* L. Int. Soc. Plant Morphol. 10, 110–115.

[B5] Audic S. Claverie J. M. (1997). The significance of digital gene expression profiles. Genome Res. 7, 986–995. 9331369 10.1101/gr.7.10.986

[B6] Baldanzi M. Pugliesi C. (1998). Selection for non-branching in castor, *Ricinus communis* L. Plant Breed. 117, 392–394. 10.1111/j.1439-0523.1998.tb01960.x

[B7] Benjamini Y. Yekutieli D. (2001). The control of the false discovery rate in multiple testing under dependency. Ann. Stat. 29, 1165–1188. 10.1214/aos/1013699998

[B8] Bentley D. R. (2006). Whole-genome re-sequencing. Curr. Opin. Genet. Dev. 16, 545–552. 10.1016/j.gde.2006.10.009 17055251

[B9] Berger S. L. (2007). The complex language of chromatin regulation during transcription. Nature 447, 407–412. 10.1038/nature05915 17522673

[B10] Bleecker A. B. Kende H. (2000). Ethylene: a gaseous signal molecule in plants. Annu. Rev. Cell Dev. Biol. 16, 1–18. 10.1146/annurev.cellbio.16.1.1 11031228

[B11] Bracale M. Caporali E. Galli M. G. Longo C. Marziani-Longo G. Rossi G. (1991). Sex determination and differentiation in *Asparagus officinalis* L. Plant Sci. 80, 67–77. 10.1016/0168-9452(91)90273-B

[B12] Brigham R. D. (1967). Inheritance of two female-sterile characters in dwarf-internode castor (*Ricinus communis* L.). Crop Sci. Soc. Am. 7, 648–650. 10.2135/cropsci1967.0011183X000700060027x

[B13] Chan A. P. Crabtree J. Zhao Q. Lorenzi H. Orvis J. Puiu D.. (2010). Draft genome sequence of the oilseed species *Ricinus communis*. Nat. Biotechnol. 28, 951–956. 10.1038/nbt.1674 20729833 PMC2945230

[B14] Chauhan S. V. S. Saxena B. K. Kinoshita T. (1987). Effect of daminozide (B9) on sex-expression and seed setting in castor bean, *Ricinus communis* L. Jpn. J. Breed. 37, 262–266. 10.1270/jsbbs1951.37.262

[B15] Chen M. L. Fu X. M. Liu J. Q. Ye T. T. Hou S. Y. Huang Y. Q.. (2012). Highly sensitive and quantitative profiling of acidic phytohormones using derivatization approach coupled with nano-LC-ESI-Q-TOF-MS analysis. J. Chromatogr. B Analyt. Technol. Biomed. Life Sci. 905, 67–74. 10.1016/j.jchromb.2012.08.005 22917596

[B16] Cheng P. C. Greyson R. I. Walden D. B. (1983). Organ initiation and the development of unisexual flowers in the tassel and ear of Zea mays. Am. J. Bot. 70, 450–462. 10.2307/2443252

[B17] Cheng X. Blumenthal R. M. (2008). Mammalian DNA methyltransferases: a structural perspective. Structure 16, 341–350. 10.1016/j.str.2008.01.004 18334209 PMC2597194

[B18] Chu V. T. Gottardo R. Raftery A. E. Bumgarner R. E. Yeung K. Y. (2008). MeV+R: using MeV as a graphical user interface for Bioconductor applications in microarray analysis. Genome Biol. 9:R118 10.1186/gb-2008-9-7-r118 18652698 PMC2530872

[B19] Conesa A. Götz S. García-Gómez J. M. Terol J. Talón M. Robles M. (2005). Blast2GO: a universal tool for annotation, visualization and analysis in functional genomics research. Bioinformatics 21, 3674–3676. 10.1093/bioinformatics/bti610 16081474

[B20] Davies P. J. (2004). Plant Hormones: Biosynthesis, Signal Transduction, Action! Dordrecht; Boston, MA; London: Kluwer academic publishers.

[B21] Dellaporta S. L. Calderon-Urrea A. (1993). Sex determination in flowering plants. Plant Cell 5, 1241–1251. 10.1105/tpc.5.10.1241 8281039 PMC160357

[B22] DeLong A. Calderon-Urrea A. Dellaporta S. L. (1993). Sex determination gene TASSELSEED2 of maize encodes a short-chain alcohol dehydrogenase required for stage-specific floral organ abortion. Cell 74, 757–768. 10.1016/0092-8674(93)90522-R 8358795

[B23] Gaganias A. A. Berg A. (1977). R.: the effect of morphactin (methyl 2-ehloro-9-hydroxyfluorene-9–carboxylate) on basipetal transport of indol-3-yl acetic acid in hypocotyl sections of Phaseolus vulgaris L. Ann. Bot. 41, 1135–1148.

[B24] Galun E. (1962). Study of the inheritance of sex expression in the cucumber. The interaction of major genes with modifying genetic and non-genetic factors. Genetica 32, 134–163. 10.1007/BF01816091

[B25] George W. L. Shifriss O. (1967). Interspersed sexuality in *Ricinus*. Genetics 57, 347–356. 17248393 10.1093/genetics/57.2.347 PMC1211731

[B26] Goff S. A. Ricke D. Lan T. H. Presting G. Wang R. Dunn M.. (2002). A draft sequence of the rice genome (*Oryza sativa* L. ssp. japonica). Science 296, 92–100. 10.1126/science.1068275 11935018

[B27] Guo Q. Q. Ma X. J. Wei S. G. Qiu D. Y. Wilson I. W. Wu P.. (2014). *De novo* transcriptome sequencing and digital gene expression analysis predict biosynthetic pathway of rhynchophylline and isorhynchophylline from Uncaria rhynchophylla, a non-model plant with potent anti-Alzheimer's properties. BMC Genomics 15:676. 10.1186/1471-2164-15-676 25112168 PMC4143583

[B28] Guo S. G. Zheng Y. Joung J. G. Liu S. Q. Zhang Z. H. Crasta O. R.. (2010). Transcriptome sequencing and comparative analysis of cucumber flowers with different sex types. BMC Genomics 11:384. 10.1186/1471-2164-11-384 20565788 PMC2897810

[B29] Halilu A. D. Aba D. A. Ogunwole J. O. (2013). Genetic variability, genetic gain and relationships of yield and yield components in castor (*Ricinus communis* L.). Res. Rev. BioSci. 7, 181–186.

[B30] Heslop-Harrison J. (1957). The experimental modification of sex expression in flowering plants. Biol. Rev. 32, 38–90. 10.1111/j.1469-185X.1957.tb01576.x

[B31] Hong L. Z. Li J. Schmidt-Küntzel A. Warren W. C. Barsh G. S. (2011). Digital gene expression for non-model organisms. Genome Res. 21, 1905–1915. 10.1101/gr.122135.111 21844123 PMC3205575

[B32] Howe E. A. Sinha R. Schlauch D. Quackenbush J. (2011). RNA-Seq analysis in MeV. Bioinformatics 27, 3209–3210. 10.1093/bioinformatics/btr490 21976420 PMC3208390

[B33] Huang S. Li R. Zhang Z. Li L. Gu X. Fan W.. (2009). The genome of the cucumber, *Cucumis sativus* L. Nat. Genet. 41, 1275–1281. 10.1038/ng.475 19881527

[B34] Jacob K. M. (1963). A trisomic male castor bean plant. J. Heredity 54, 292–296.

[B35] Jacob K. M. Atsmon D. (1965). Sex inheritance in Ricinus communis L.: evidence for a genetic change during the ontogeny of female sex reversals. Genetica 36, 253–259. 10.1007/BF01557157 5884409

[B36] Jeong G. T. Park D. H. (2009). Optimization of biodiesel production from castor oil using response surface methodology. Appl. Biochem. Biotech. 156, 431–441. 10.1007/s12010-008-8468-9 19089650

[B37] Kaplan B. Davydov O. Knight H. Galon Y. Knight M. R. Fluhr R.. (2006). Rapid transcriptome changes induced by cytosolic Ca2+ transients reveal ABRE-related sequences as Ca2+-responsive cis elements in Arabidopsis. Plant Cell 18, 2733–2748. 10.1105/tpc.106.042713 16980540 PMC1626612

[B38] Kater M. M. Franken J. Carney K. J. Colombo L. Angenent G. C. (2001). Sex determination in the monoecious species cucumber is confined to specific floral whorls. Plant Cell 13, 481–493. 10.1105/tpc.13.3.481 11251091 PMC135508

[B39] Kumar N. Rao P. (1980). Influence of kinetin and morphactin on changes in sex expression, carbohydrate and nitrogen fractions in castor (*Ricinus communis* L.). Proc. Plant Sci. 89, 457–464.

[B40] Kumar N. R. Rao P. G. (1981). Activity of auxin like substances in relation to feminization of castor bean (*Ricinus communis* L.) influenced by kinetin and morphactin. Turrialba 31, 201–208.

[B41] Li R. Q. Yu C. Li Y. R. Lam T. W. Yiu S. M. Kristiansen K.. (2009a). SOAP2: an improved ultrafast tool for short read alignment. Bioinformatics 25, 1966–1967. 10.1093/bioinformatics/btp336 19497933

[B42] Li Z. Huang S. W. Liu S. Q. Pan J. S. Zhang Z. H. Tao Q. Y.. (2009b). Molecular isolation of the M gene suggests that a conserved-residue conversion induces the formation of bisexual flowers in cucumber plants. Genetics 182, 1381–1385. 10.1534/genetics.109.104737 19474195 PMC2728875

[B43] Lorrain S. Vailleau F. Balagué C. Roby D. (2003). Lesion mimic mutants: keys for deciphering cell death and defense pathways in plants. Trends Plant Sci. 8, 263–271. 10.1016/S1360-1385(03)00108-0 12818660

[B44] Malepszy S. Niemirowicz-Szczytt K. (1991). Sex determination in cucumber (*Cucumis sativus*) as a model system for molecular biology. Plant Sci. 80, 39–47. 10.1016/0168-9452(91)90271-9

[B45] Martin A. Troadec C. Boualem A. Rajab M. Fernandez R. Morin H.. (2009). A transposon-induced epigenetic change leads to sex determination in melon. Nature 461, 1135–1138. 10.1038/nature08498 19847267

[B46] McIntyre L. M. Lopiano K. K. Morse A. M. Amin V. Oberg A. L. Young L. J.. (2011). RNA-seq: technical variability and sampling. BMC Genomics 12:293. 10.1186/1471-2164-12-293 21645359 PMC3141664

[B47] Mibus H. Tatlioglu T. (2004). Molecular characterization and isolation of the F/f gene for femaleness in cucumber (*Cucumis sativus* L.). Theor. Appl. Genet. 109, 1669–1676. 10.1007/s00122-004-1793-7 15490106

[B48] Mohan Ram H. Y. Sett R. (1980). Induction of male flowers in a pistillate line of *Ricinus communis* L. by silver and cobalt ions. Planta 149, 413–415. 24306381 10.1007/BF00571179

[B49] Morrissy A. S. Morin R. D. Delaney A. Zeng T. McDonald H. Jones S.. (2009). Next-generation tag sequencing for cancer gene expression profiling. Genome Res. 19, 1825–1835. 10.1101/gr.094482.109 19541910 PMC2765282

[B50] Oshlack A. Robinson M. D. Young M. D. (2010). From RNA-seq reads to differential expression results. Genome Biol. 11:220. 10.1186/gb-2010-11-12-220 21176179 PMC3046478

[B51] Peng J. Richards D. E. Hartley N. M. Murphy G. P. Devos K. M. Flintham J. E.. (1999). ‘Green revolution’ genes encode mutant gibberellin response modulators. Nature 400, 256–261. 10.1038/22307 10421366

[B52] Philipos H. Narayanaswamy S. (1976). Induced potential for male sex expression inRicinus communis L. by ethyl hydrogen-1-propylphosphonate. Protoplasma 87, 71–77. 10.1007/BF01623959

[B53] Pranavi B. Sitaram G. Yamini K. N. Dinesh Kumar V. (2011). Development of EST–SSR markers in castor bean (*Ricinus communis* L.) and their utilization for genetic purity testing of hybrids. Genome 54, 684–691. 10.1139/g11-033 21848404

[B54] Ramsey S. A. Klemm S. L. Zak D. E. Kennedy K. A. Thorsson V. Li B.. (2008). Uncovering a macrophage transcriptional program by integrating evidence from motif scanning and expression dynamics. PLoS Comput. Biol. 4:e1000021. 10.1371/journal.pcbi.1000021 18369420 PMC2265556

[B55] Rao P. G. (1969). Sex expression in *Ricinus communis* L. Sci. Cult. 35, 326–327.

[B56] Rkey V. A. (1978). M.: Experimental Studies on Effects of Plant Growth Regulators on Vegetative Morphology and Floral Morphogenesis in Ricinus communis L. Ph.D dissertation (Agronomy), Kaupur University.

[B57] Savy Filho A. (2005). Castor bean breeding, in Improvement of Cultivated Species, ed Borém A. (Viçosa: Federal University of Viçosa).

[B58] Shifriss O. (1956). Sex instability in ricinus. Genetics 41, 265–280. 17247626 10.1093/genetics/41.2.265 PMC1209780

[B59] Shifriss O. (1960). Conventional and unconventional systems controlling sex variations in *Ricinus*. J. Genet. 57, 361–388. 10.1007/BF02987242

[B60] Shifriss O. (1961). Gibberellin as Sex Regulator in *Ricinus communis*. Science 133, 2061–2062. 10.1126/science.133.3470.2061 17814561

[B61] Tan M. Yan M. Wang L. Yan X. (2011). Effect of chemical treatments on sex expression of *Ricinus communis*. Chin. Agr. Sci. Bull. 27, 164–169.

[B62] Tanurdzic M. Banks J. A. (2004). Sex-determining mechanisms in land plants. Plant Cell 16 Suppl., S61–S71. 10.1105/tpc.016667 15084718 PMC2643385

[B63] 't Hoen P. A. C. Ariyurek Y. Thygesen H. H. Vreugdenhil E. Vossen R. H. A. M. de Menezes R. X.. (2008). Deep sequencing-based expression analysis shows major advances in robustness, resolution and inter-lab portability over five microarray platforms. Nucleic Acids Res. 36:e141. 10.1093/nar/gkn705 18927111 PMC2588528

[B64] Thomson K. S. Leopold A. C. (1974). *In-vitro* binding of morphactins and 1-N-naphthylphthalamic acid in corn coleoptiles and their effects on auxin transport. Planta 115, 259–270. 10.1007/BF00390522 24458888

[B65] Tian D. Q. Pan X. Y. Yu Y. M. Wang W. Y. Zhang F. Ge Y. Y.. (2013). *De novo* characterization of the Anthurium transcriptome and analysis of its digital gene expression under cold stress. BMC Genomics 14:827. 10.1186/1471-2164-14-827 24267953 PMC4046746

[B66] Trebitsh T. Rudich J. Riov J. (1987). Auxin, biosynthesis of ethylene and sex expression in cucumber (*Cucumis sativus*). Plant Growth Regul. 5, 105–113. 10.1007/BF00024738

[B67] Trebitsh T. Staub J. E. O'Neill S. D. (1997). Identification of a 1-aminocyclopropane-1-carboxylic acid synthase gene linked to the female (F) locus that enhances female sex expression in cucumber. Plant Physiol. 113, 987–995. 9085580 10.1104/pp.113.3.987 PMC158220

[B68] Varkey M. Nigam R. K. (1982). A gradual reduction of female structures in the pistillate flowers of Ricinus communis L. (castor-bean) with chlorflurenol (morphactin). Biol. Plantarum. 24, 152–154. 10.1007/BF02902863

[B69] Veit B. Schmidt R. J. Hake S. Yanofsky M. F. (1993). Maize floral development: new genes and old mutants. Plant Cell 5, 1205–1215. 10.1105/tpc.5.10.1205 12271023 PMC160354

[B70] Wang L. Tan M. Yan M. Wang L. Yan X. (2012). Initial research on inflorescence characteristics and flower bud differentiation of *Ricinus communis* L. Chin. J. Oil Crop Sci. 34, 544–550.

[B71] Wang X. Wang H. Wang J. Sun R. Wu J. Liu S.. (2011). The genome of the mesopolyploid crop species *Brassica rapa*. Nat. Genet. 43, 1035–1039. 10.1038/ng.919 21873998

[B72] Wei M. M. Song M. Z. Fan S. L. Yu S. X. (2013). Transcriptomic analysis of differentially expressed genes during anther development in genetic male sterile and wild type cotton by digital gene-expression profiling. BMC Genomics 14:97. 10.1186/1471-2164-14-97 23402279 PMC3599889

[B73] Wittwer S. H. Hillyer I. G. (1954). Chemical induction of male sterility in cucurbits. Science 120, 893–894. 10.1126/science.120.3126.893 17830772

[B74] Wu S. Liao G. Guo C. (1998). Floral Development in *Ricinus Communis* L. (Euphorbiaceae). Biol. Bull. NTNU 33, 105–113.

[B75] Wu T. Qin Z. W. Zhou X. Y. Feng Z. Du Y. L. (2010). Transcriptome profile analysis of floral sex determination in cucumber. J. Plant Physiol. 167, 905–913. 10.1016/j.jplph.2010.02.004 20303197

[B76] Xu Q. Chen L. L. Ruan X. Chen D. Zhu A. Chen C.. (2013). The draft genome of sweet orange (*Citrus sinensis*). Nat. Genet. 45, 59–66. 10.1038/ng.2472 23179022

[B77] Yamasaki S. Fujii N. Takahashi H. (2000). The ethylene-regulated expression of CS-ETR2 and CS-ERS genes in cucumber plants and their possible involvement with sex expression in flowers. Plant Cell Physiol. 41, 608–616. 10.1093/pcp/41.5.608 10929944

[B78] Ye D. Oliveira M. Veuskens J. Wu Y. Installe P. Hinnisdaels S. (1991). Sex determination in the dioecious Melandrium. The X/Y chromosome system allows complementary cloning strategies. Plant Sci. 80, 93–106. 10.1016/0168-9452(91)90275-D

[B79] Ye J. Fang L. Zheng H. K. Zhang Y. Chen J. Zhang Z. J.. (2006). WEGO: a web tool for plotting GO annotations. Nucleic Acids Res. 34, 293–297. 10.1093/nar/gkl031 16845012 PMC1538768

[B80] Yin T. J. Quinn J. A. (1995). Tests of a mechanistic model of one hormone regulating both sexes in *Cucumis sativus* (Cucurbitaceae). Am. J. Bot. 82, 1537–1546. 10.2307/2446182

[B81] Yu J. Hu S. Wang J. Wong G. K. Li S. Liu B.. (2002). A draft sequence of the rice genome (*Oryza sativa* L. ssp. indica). Science 296, 79–92. 10.1126/science.1068037 11935017

[B82] Yu Y. Huang W. Chen H. Wu G. Yuan H. Song X.. (2014). Identification of differentially expressed genes in flax (*Linum usitatissimum* L.) under saline-alkaline stress by digital gene expression. Gene. 549, 113–122. 10.1016/j.gene.2014.07.053 25058012

[B83] Zhang S. P. Xiao Y. N. Zhao J. R. Wang F. G. Zheng Y. L. (2013). Digital gene expression analysis of early root infection resistance to Sporisorium reilianum f. sp zeae in maize. Mol. Genet. Genomics 288, 21–37. 10.1007/s00438-012-0727-3 23196693

[B84] Zhao W. Xia W. Li J. Sheng S. Lei L. Zhao S. (2014). Transcriptome profiling and digital gene expression analysis of Fallopia multiflora to discover putative genes involved in the biosynthesis of 2,3,5,4′-tetrahydroxy stilbene-2-O-β-d-glucoside. Gene. 547, 126–135. 10.1016/j.gene.2014.06.041 24967942

