# Supplementary figures and images for: Transcriptomic Analysis for Different Sex Types of Ricinus communis L. during Development from Apical Buds to Inflorescences by Digital Gene Expression Profiling
Source: Front Plant Sci. 2016 Feb 12;6:1208. doi: 10.3389/fpls.2015.01208 (PMC4751274; doi:10.3389/fpls.2015.01208)

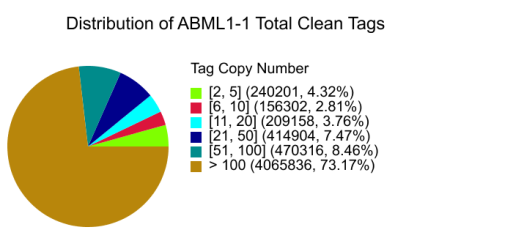

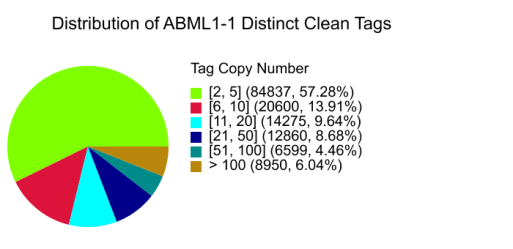

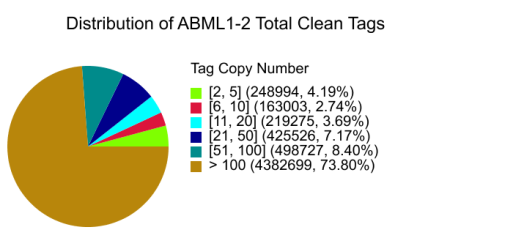

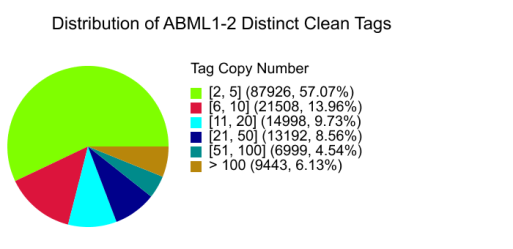

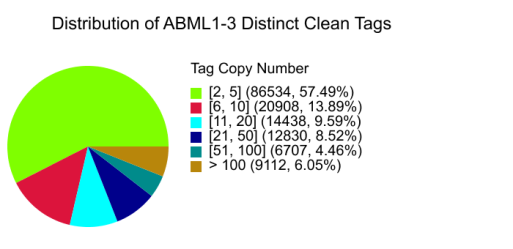

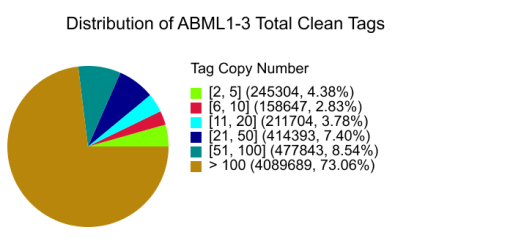

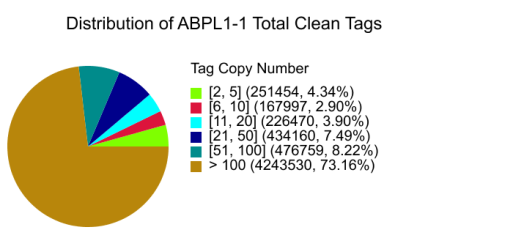

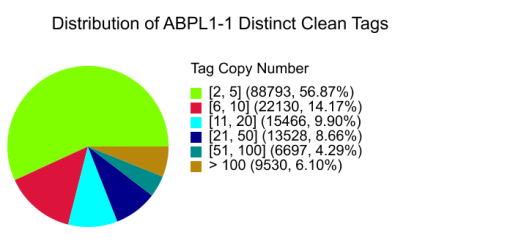

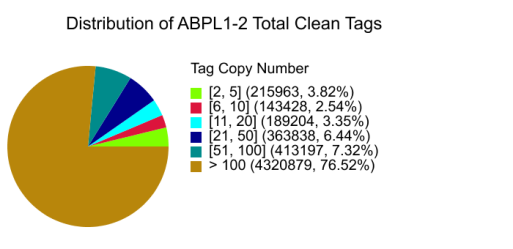

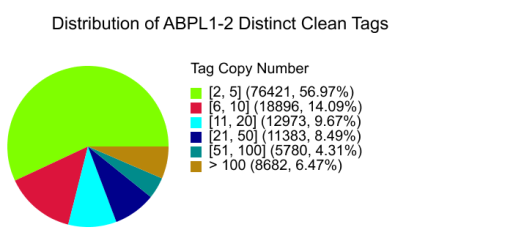

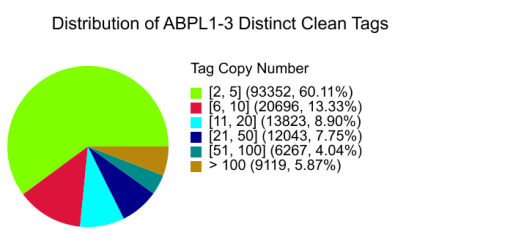

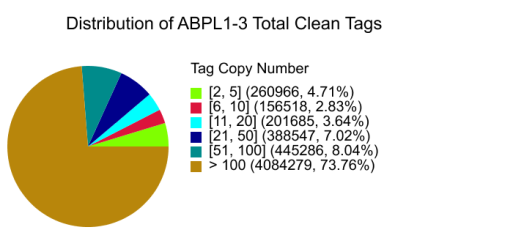

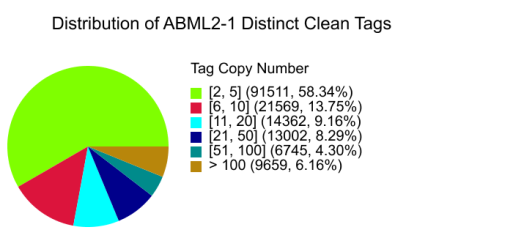

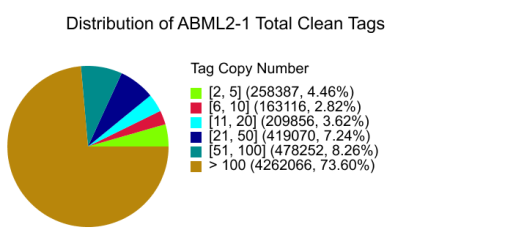

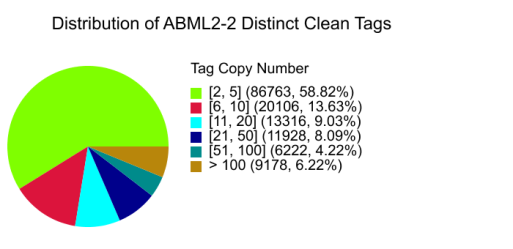

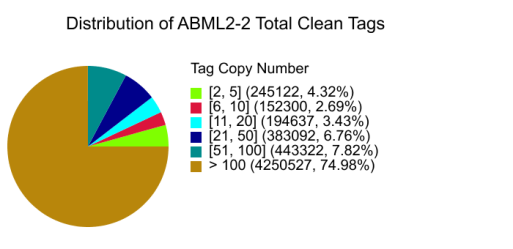

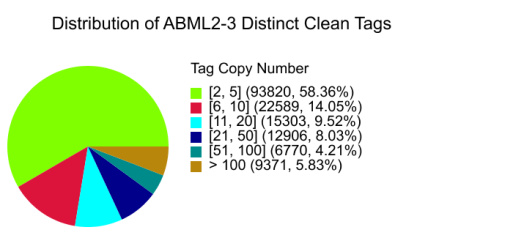

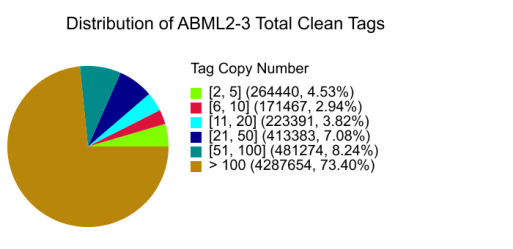

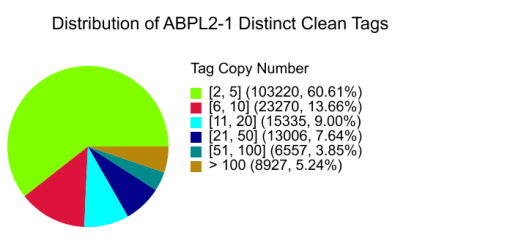

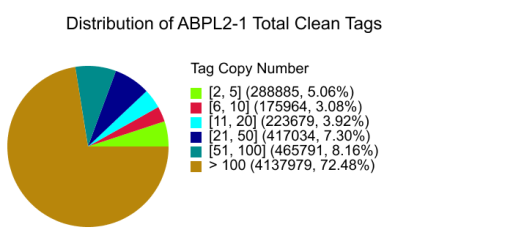

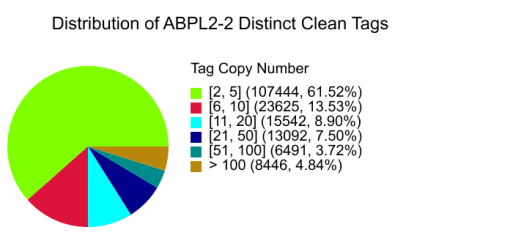

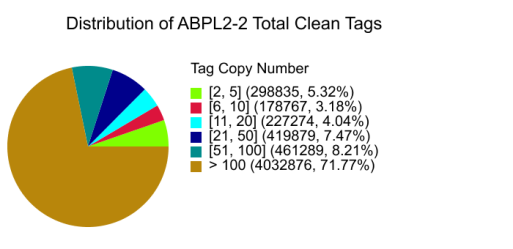

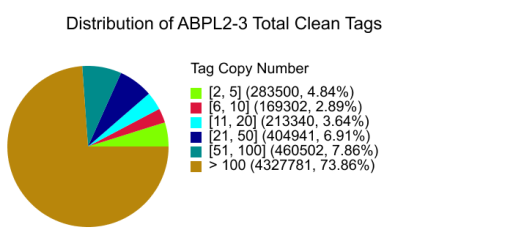


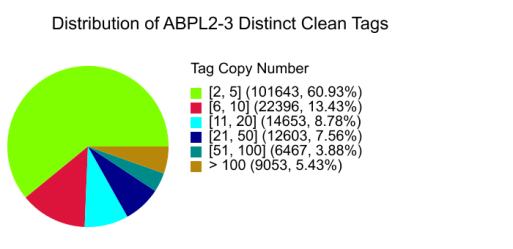


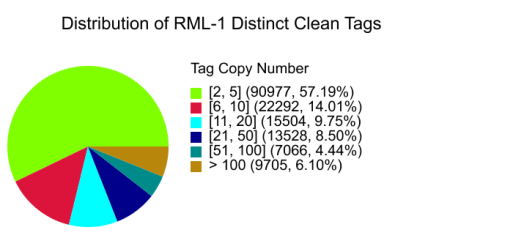

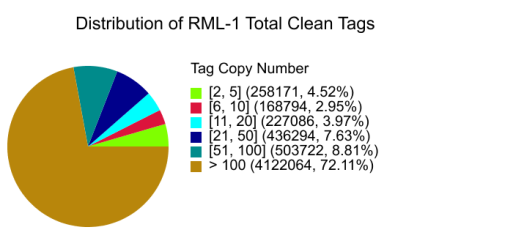

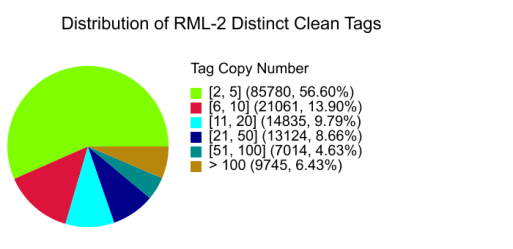

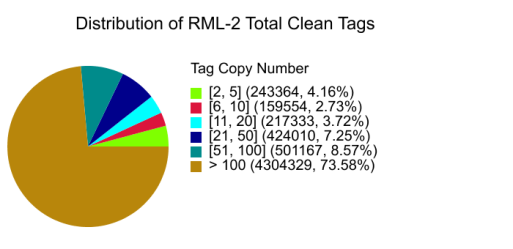

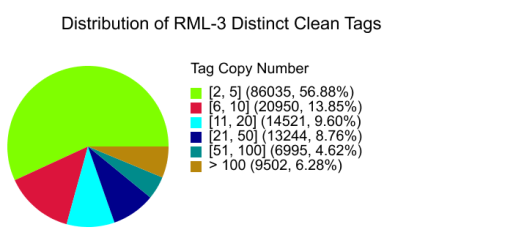

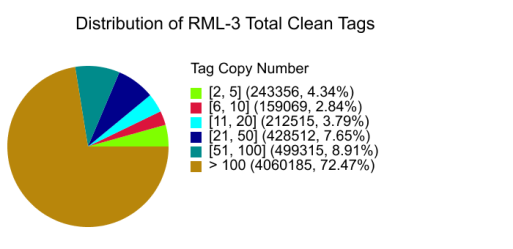

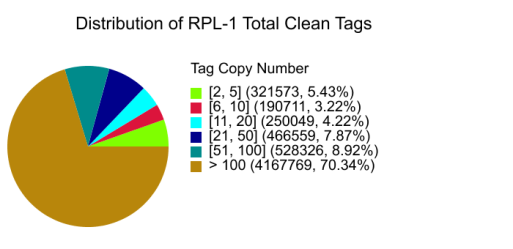

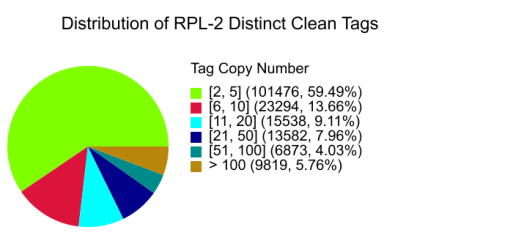

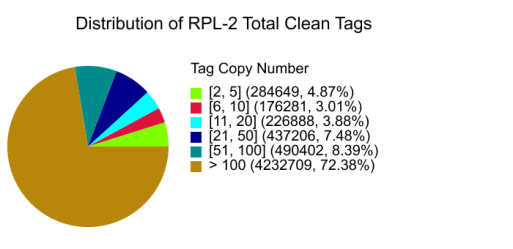

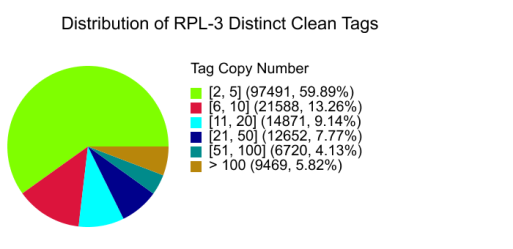

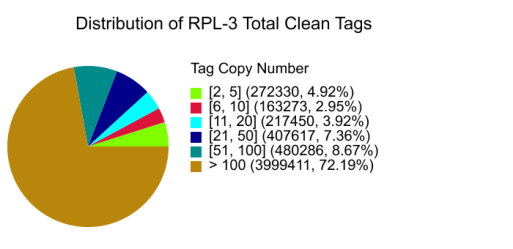

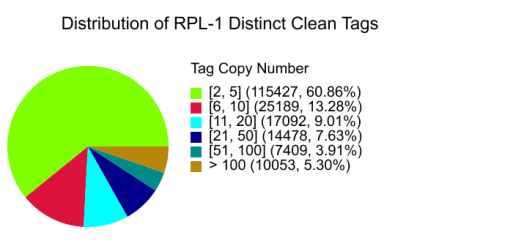

Supplement: Supplementary file 2 [file DataSheet2.DOCX]

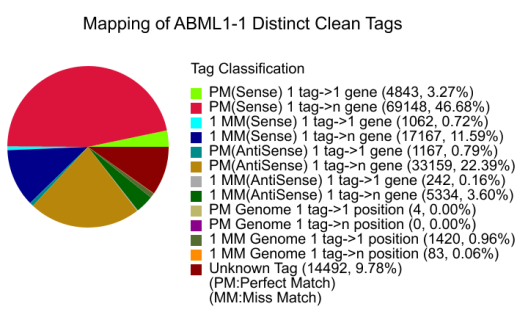

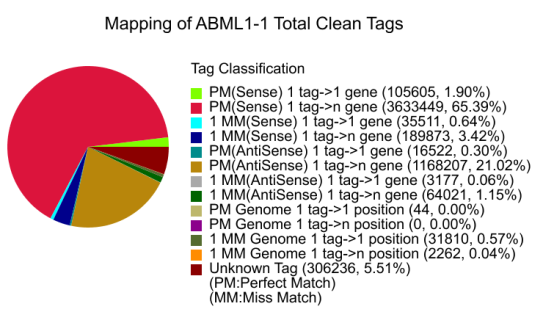


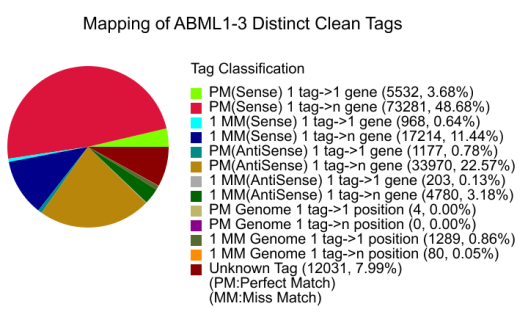

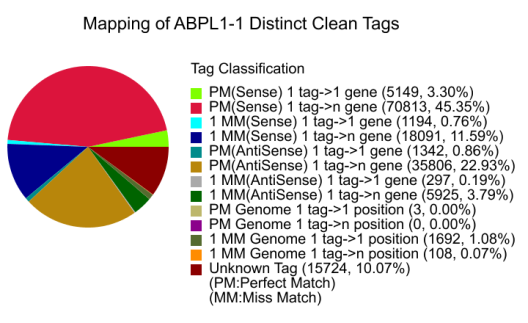

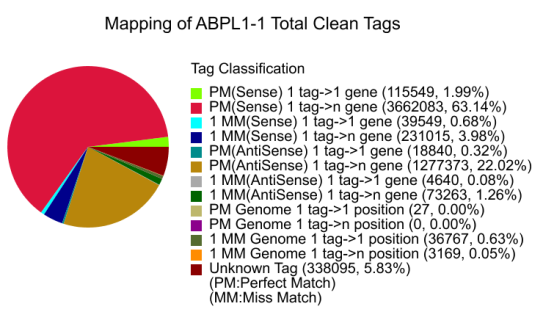

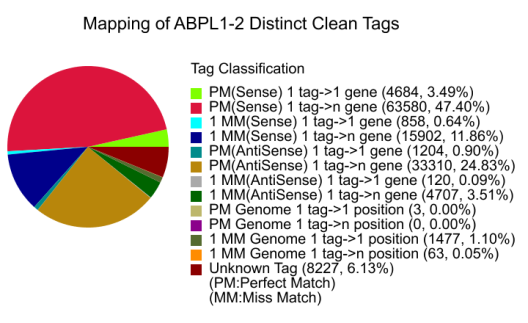

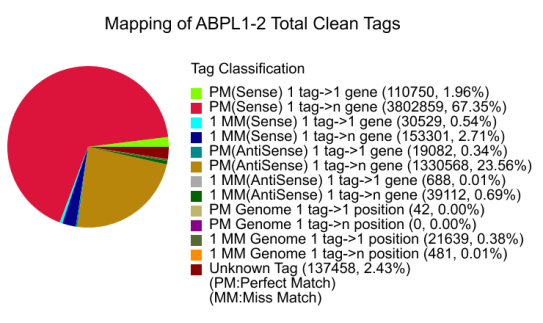

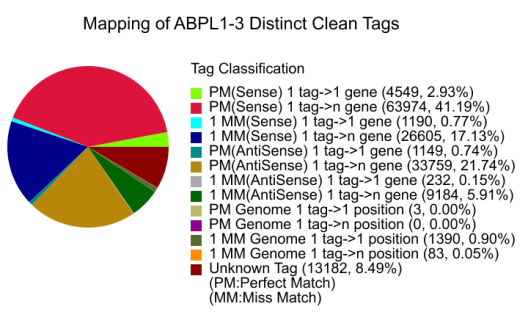

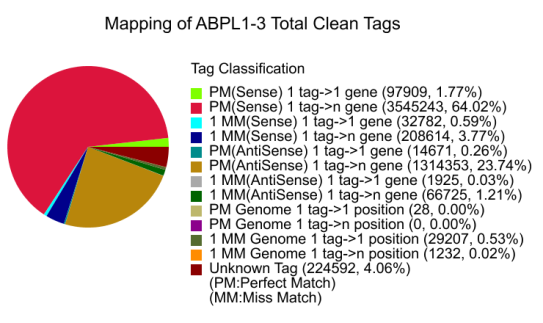

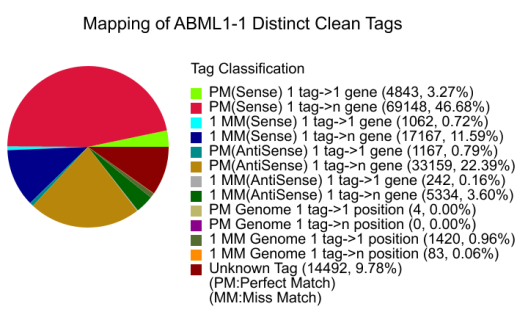

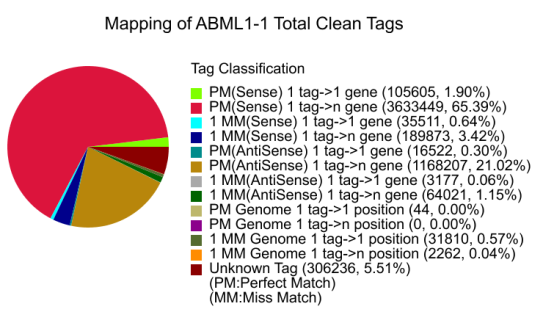

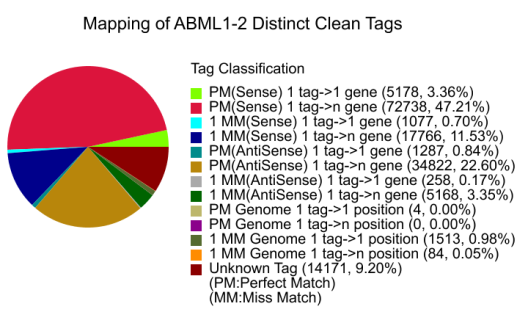

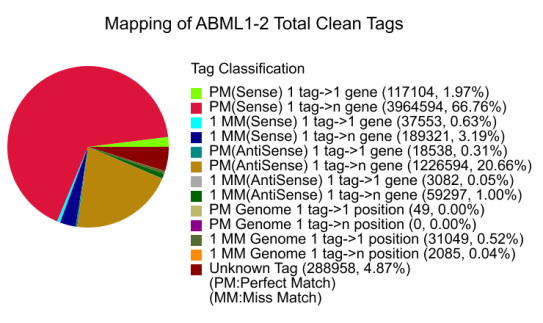

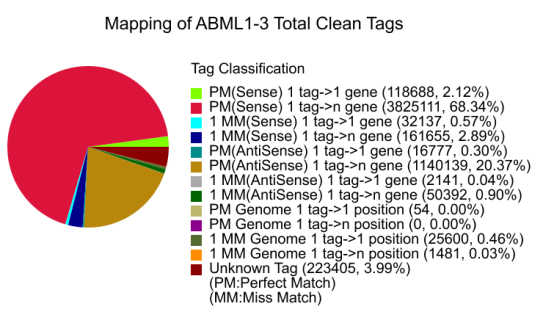


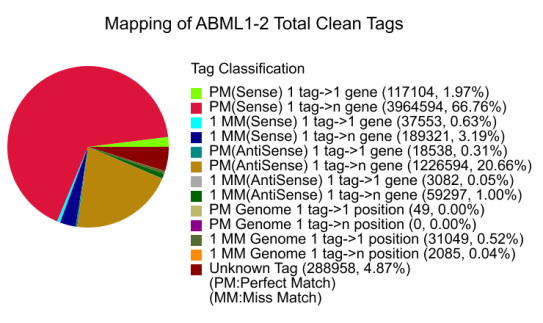

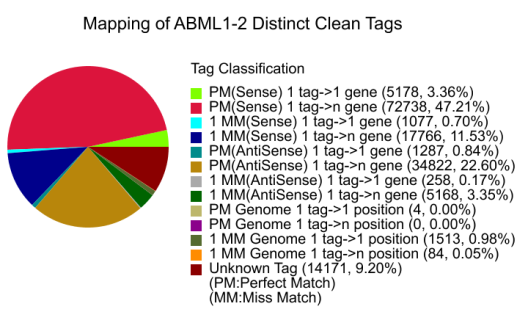


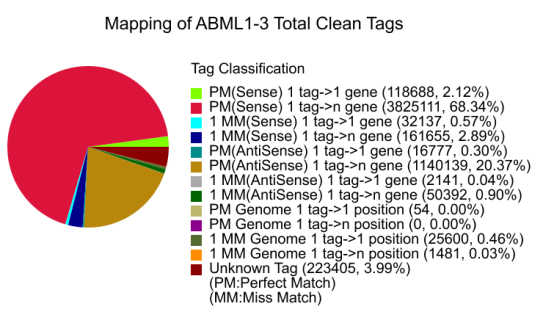


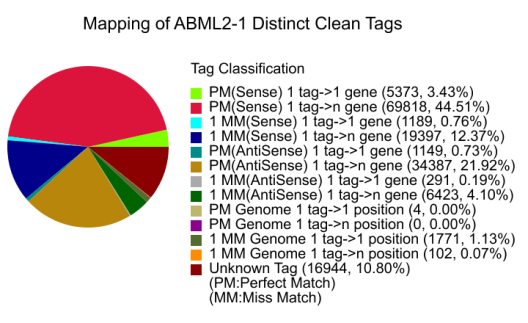

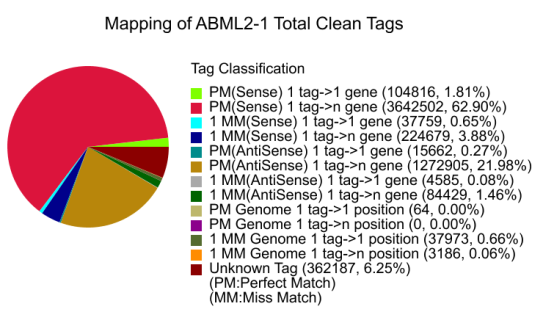

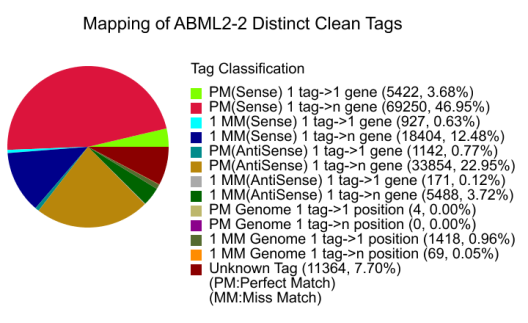

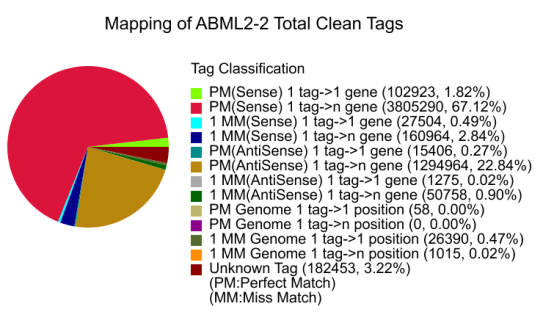

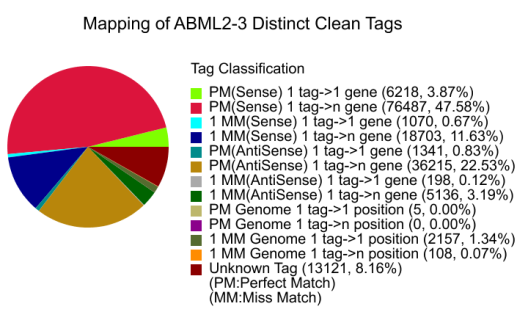

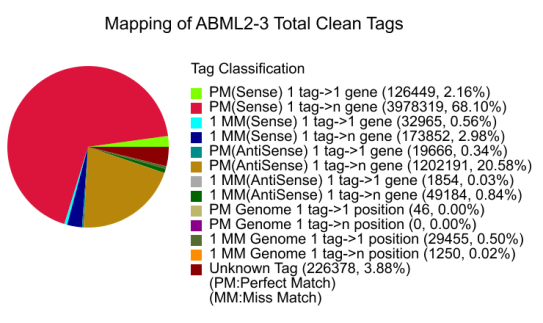

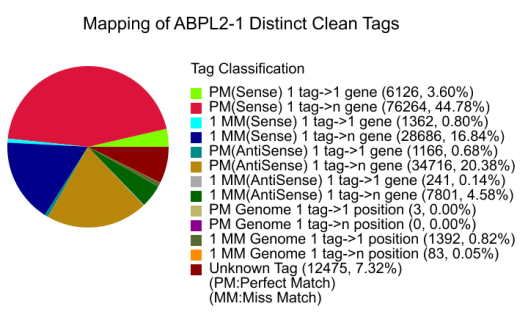

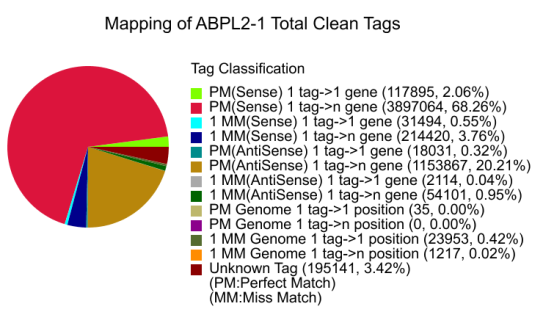

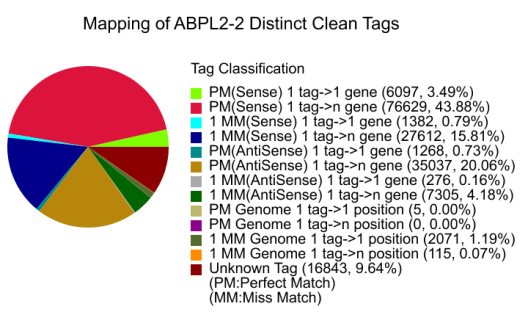

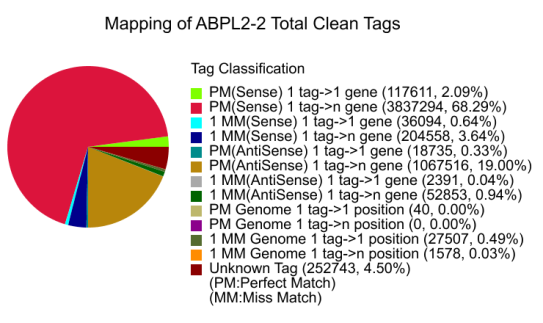

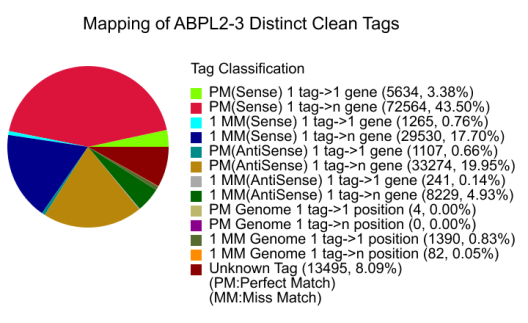

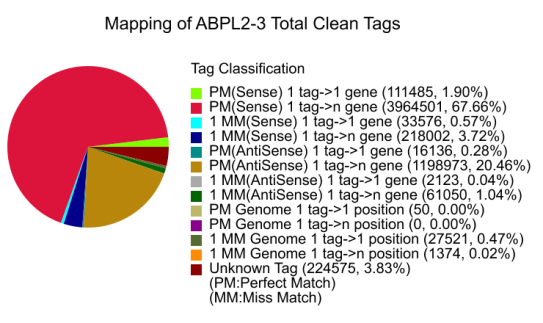

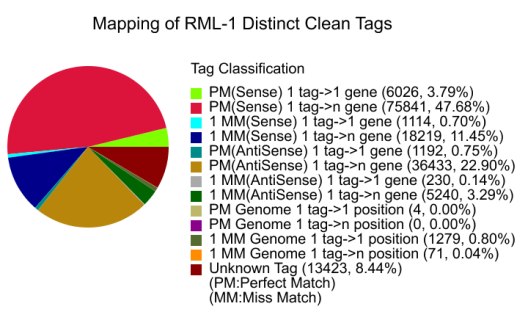

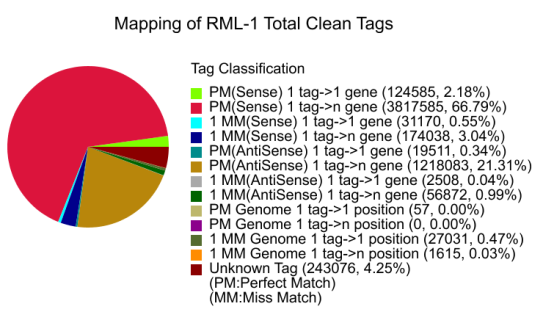

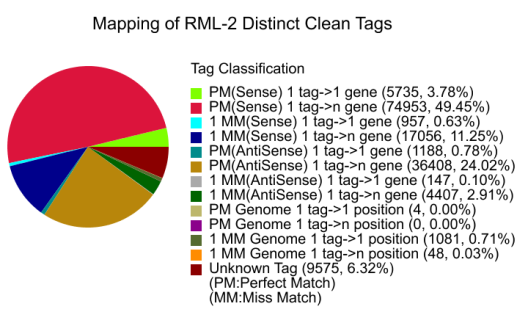

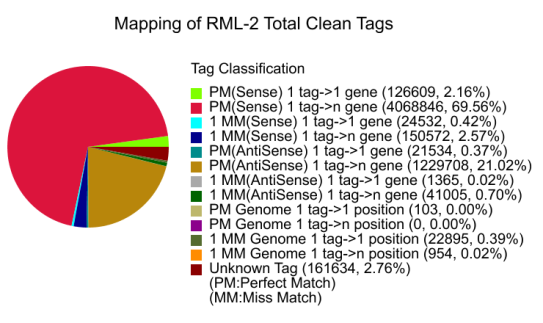

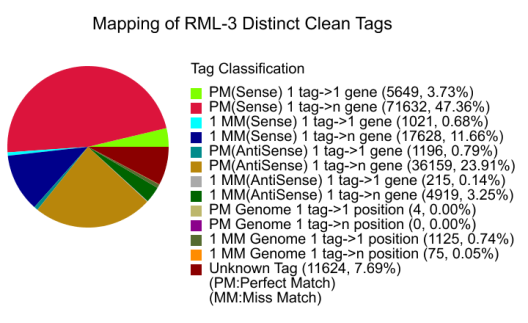

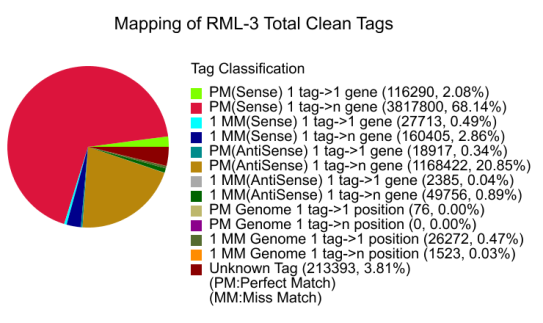

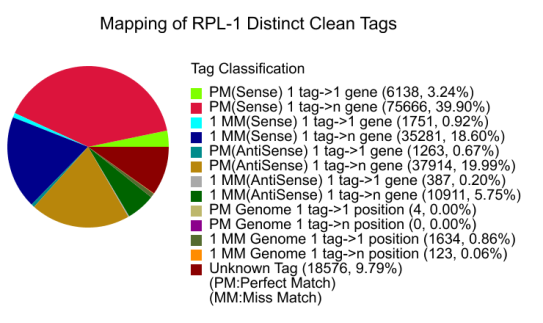

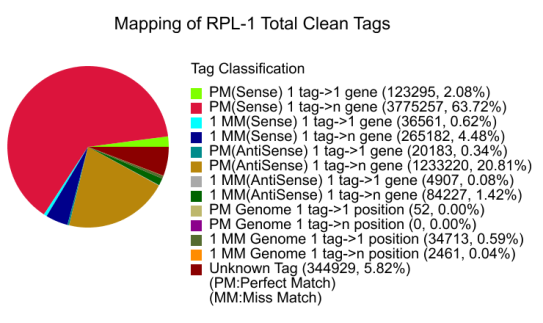

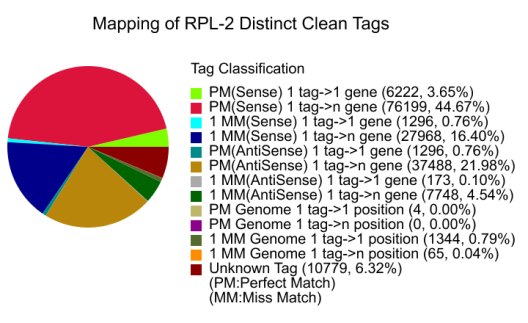

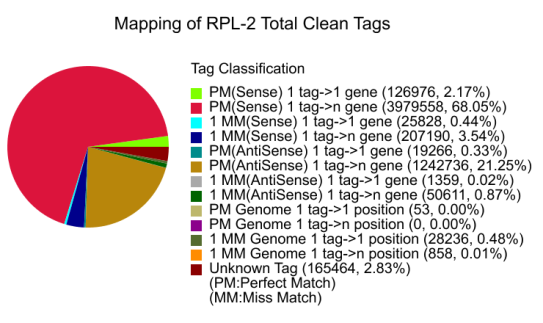

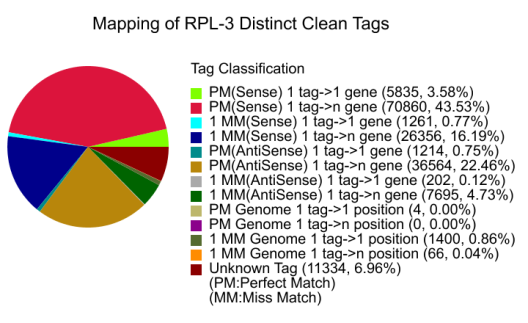

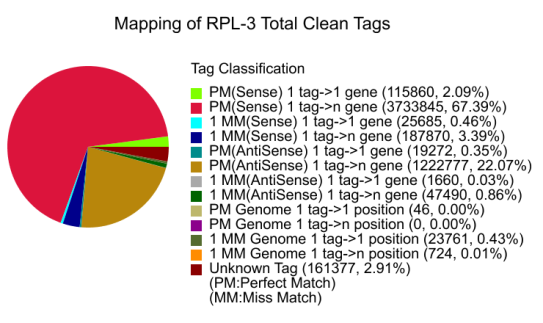

Supplement: Supplementary file 4 [file DataSheet4.DOCX]

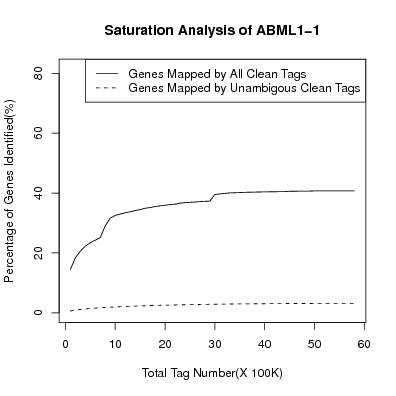

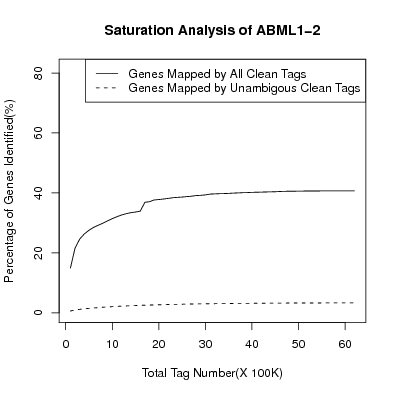

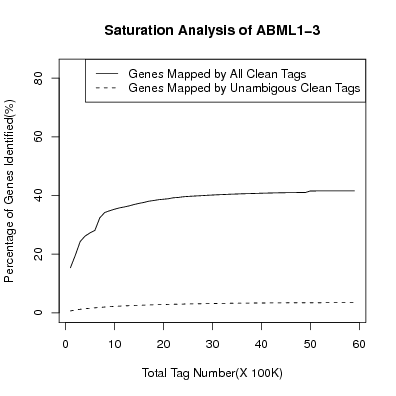

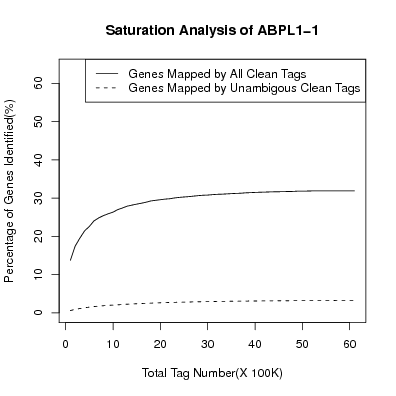

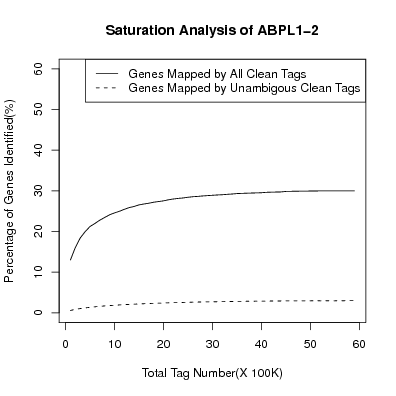

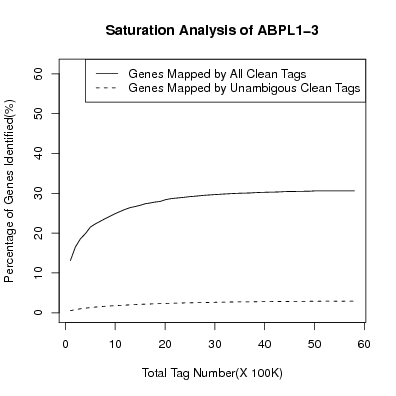

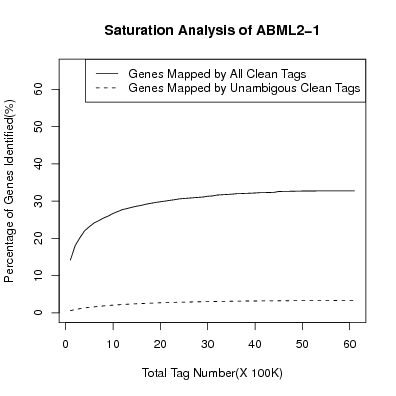

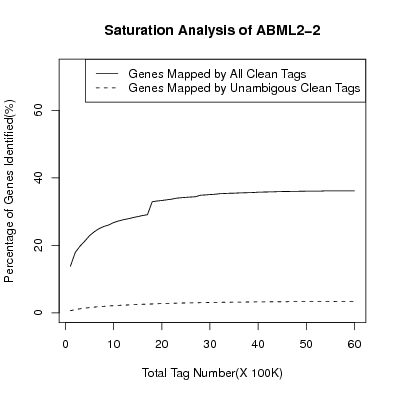

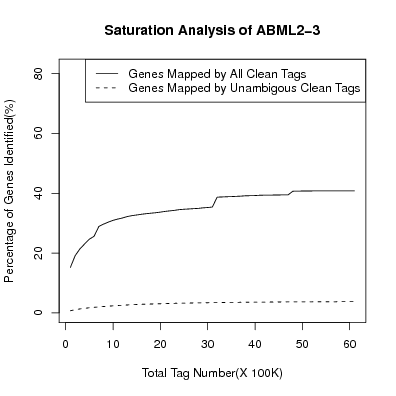

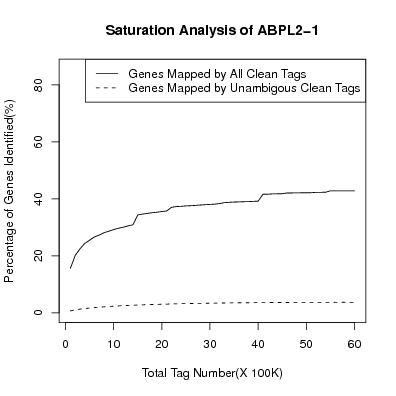

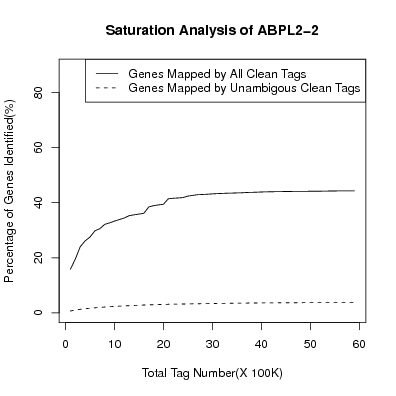

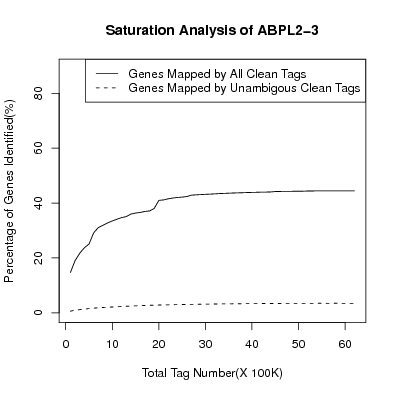

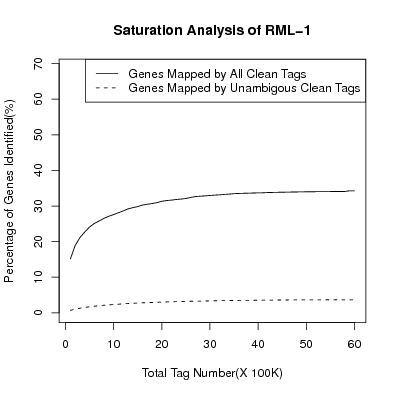

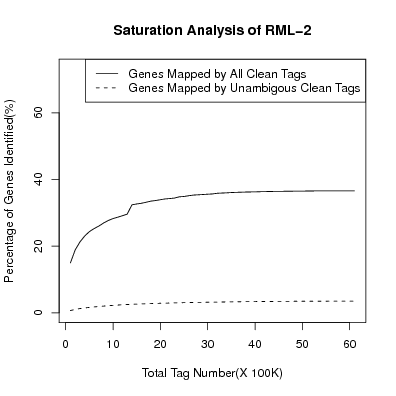

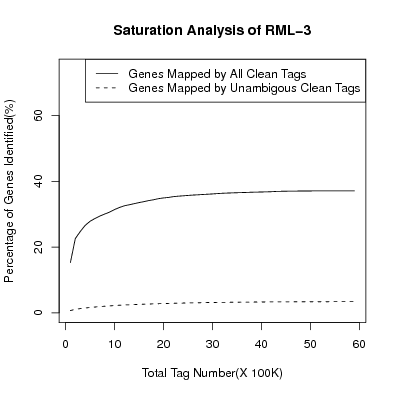

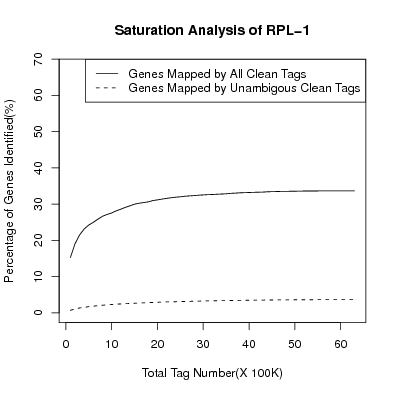

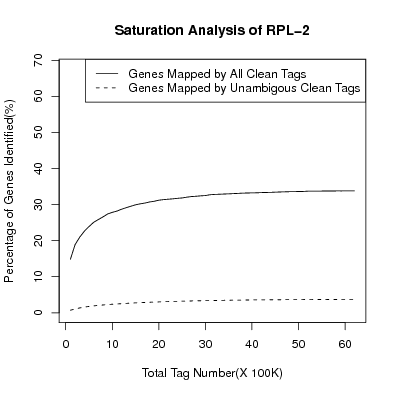

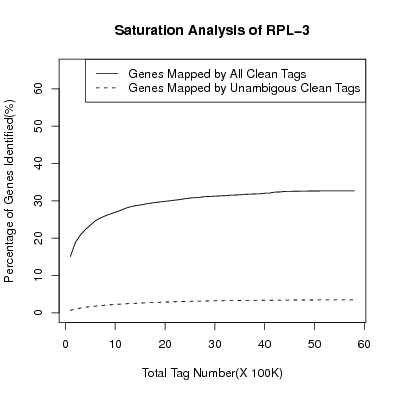

Supplement: Supplementary file 5 [file DataSheet5.DOCX]
