# Supplementary material for: Transcriptomic Analysis for Different Sex Types of Ricinus communis L. during Development from Apical Buds to Inflorescences by Digital Gene Expression Profiling
Source: Front Plant Sci. 2016 Feb 12;6:1208. doi: 10.3389/fpls.2015.01208 (PMC4751274; doi:10.3389/fpls.2015.01208)
Supplement: Supplementary file 8 [file DataSheet8.DOCX]

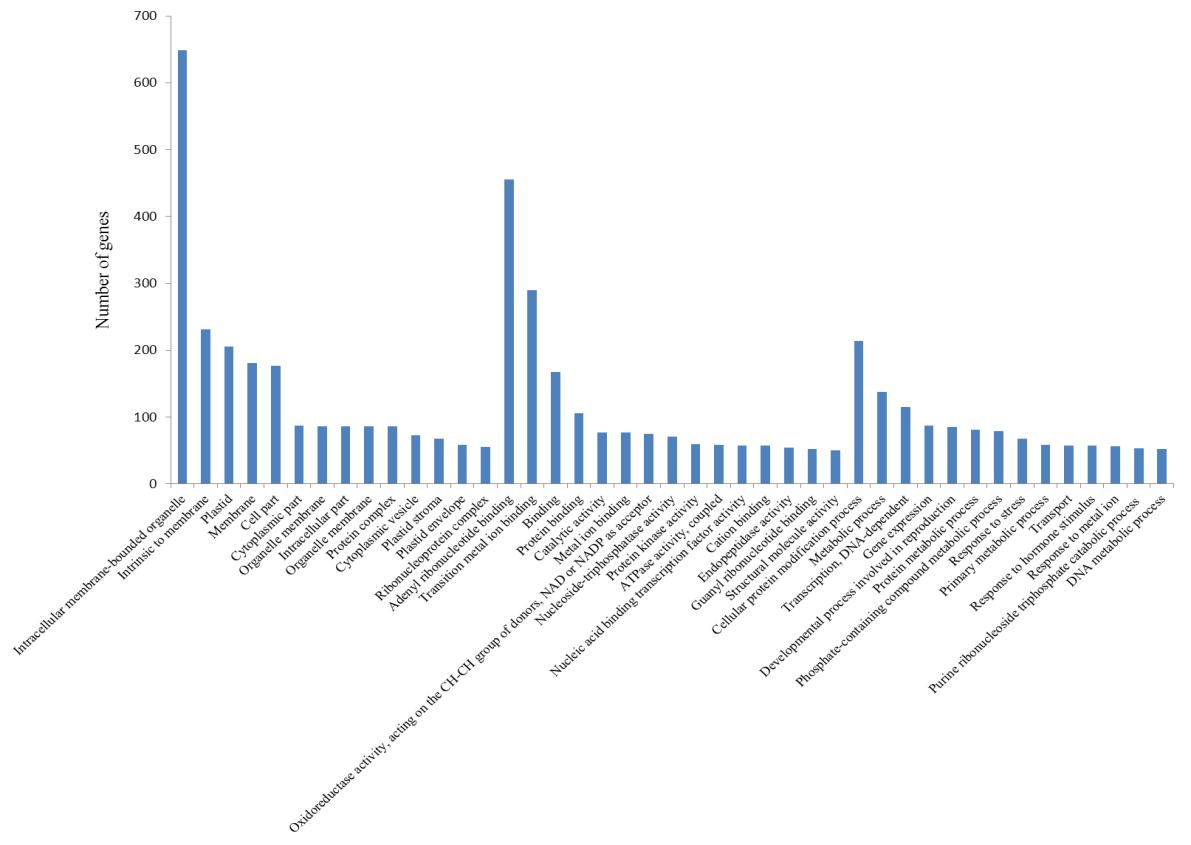


Cellular Component

Molecular Function

Biological Process

**Histogram of main gene ontology classification of unambiguously mapped genes in all libraries.** Unambiguously mapped genes were assigned to three main categories: cellular component, molecular function, and biological process. Here, only the main categories (i.e., categories containing ≥50 genes) are listed; 108 subcategories of cellular component including 622 genes, 414 subcategories of molecular function including 2,219 genes, and 670 subcategories of biological process including 2,592 genes were omitted.

**ABML1 VS ABML2**


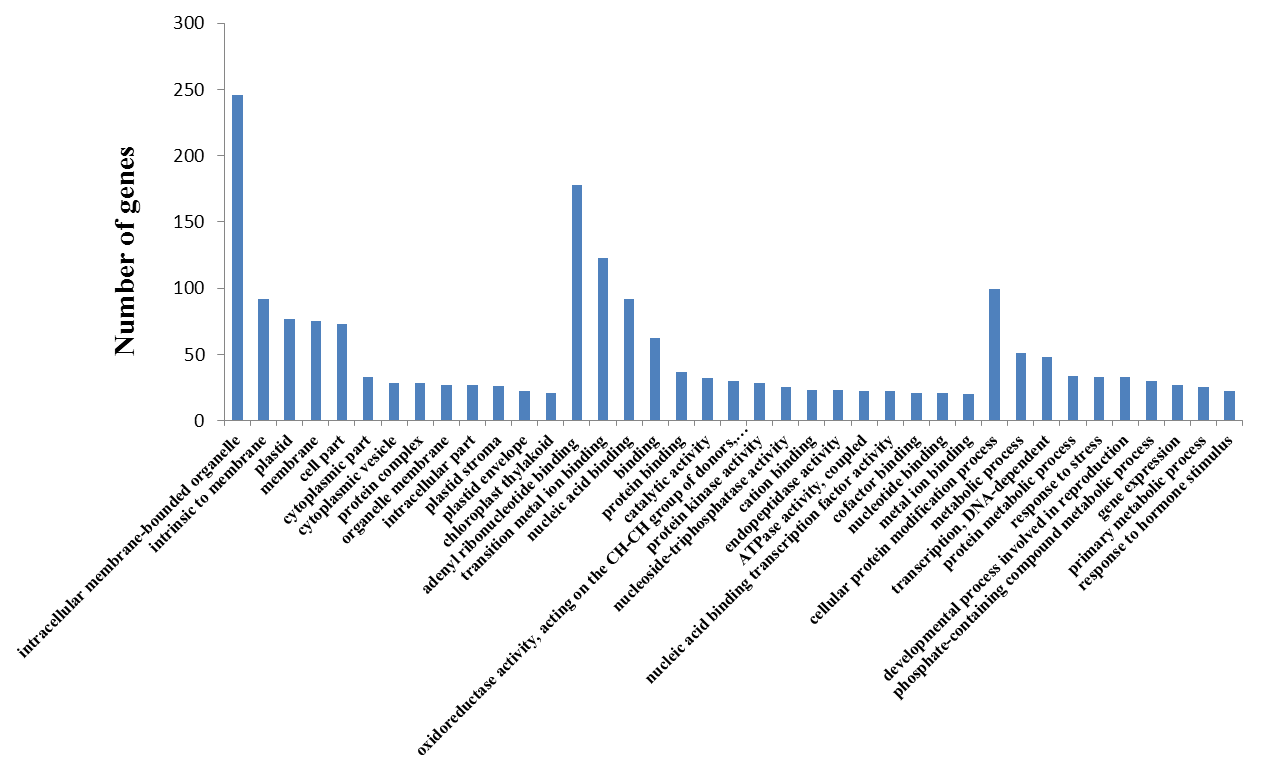


Cellular Component

Molecular Function

Biological Process

**Histogram presentation of gene ontology classification of ABML1 VS ABML2.** Here only the main categories (the number of genes in the category ≥ 20) were listed; other 83 sorts including 261 genes in cellular component, other 277 classifications containing 872 genes in molecular function and 438 categories comprised by 1066 genes in biological process were omitted.


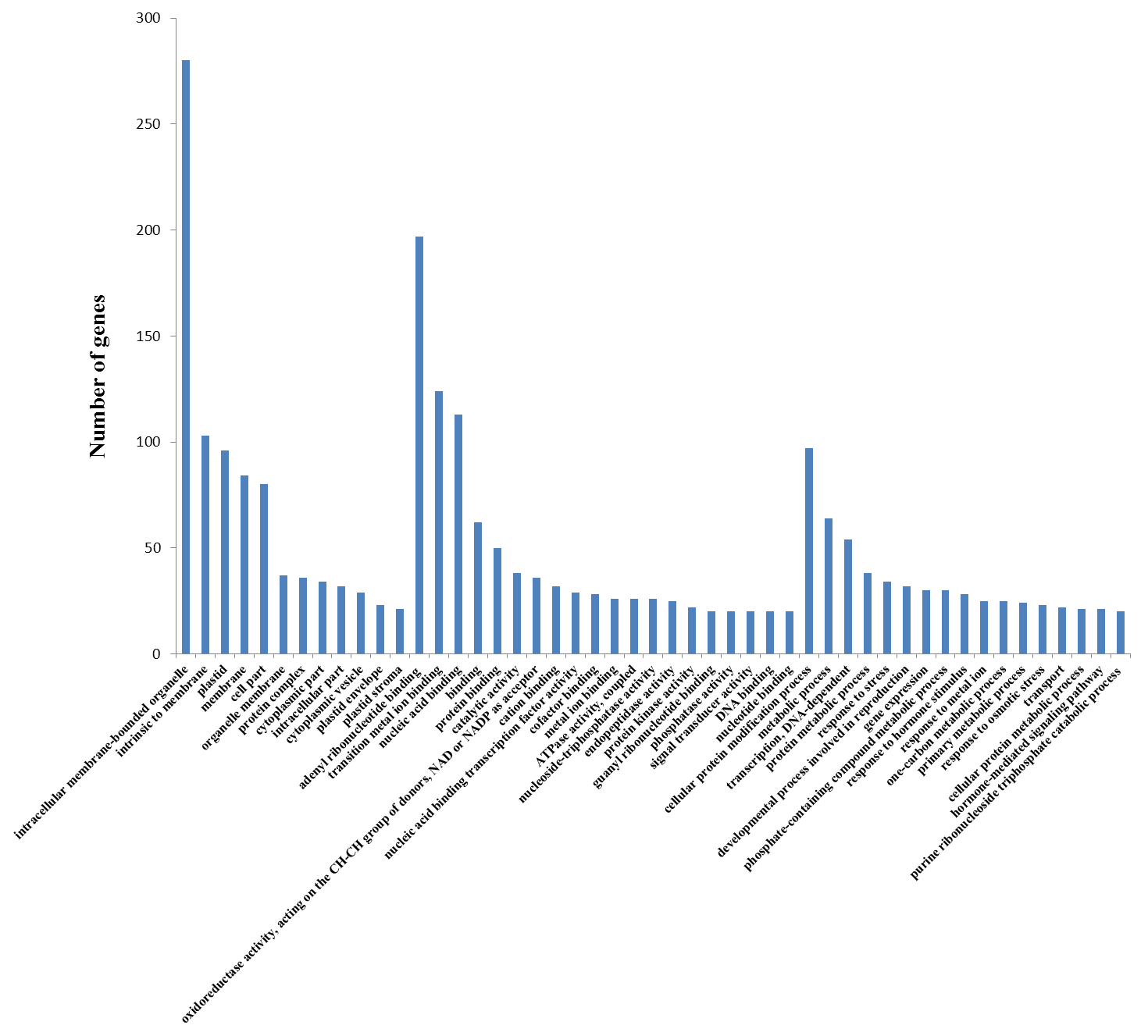


Cellular Component

Molecular Function

Biological Process

**Gene ontology classification of ABML2 VS RML.** Only the main categories (the number of genes in the category ≥ 20) were listed; other 91 sorts including 309 genes in cellular component, other 292 classifications containing 895 genes in molecular function and 455 categories comprised by 1031 genes in biological process were not shown.

**ABML2 VS RML**


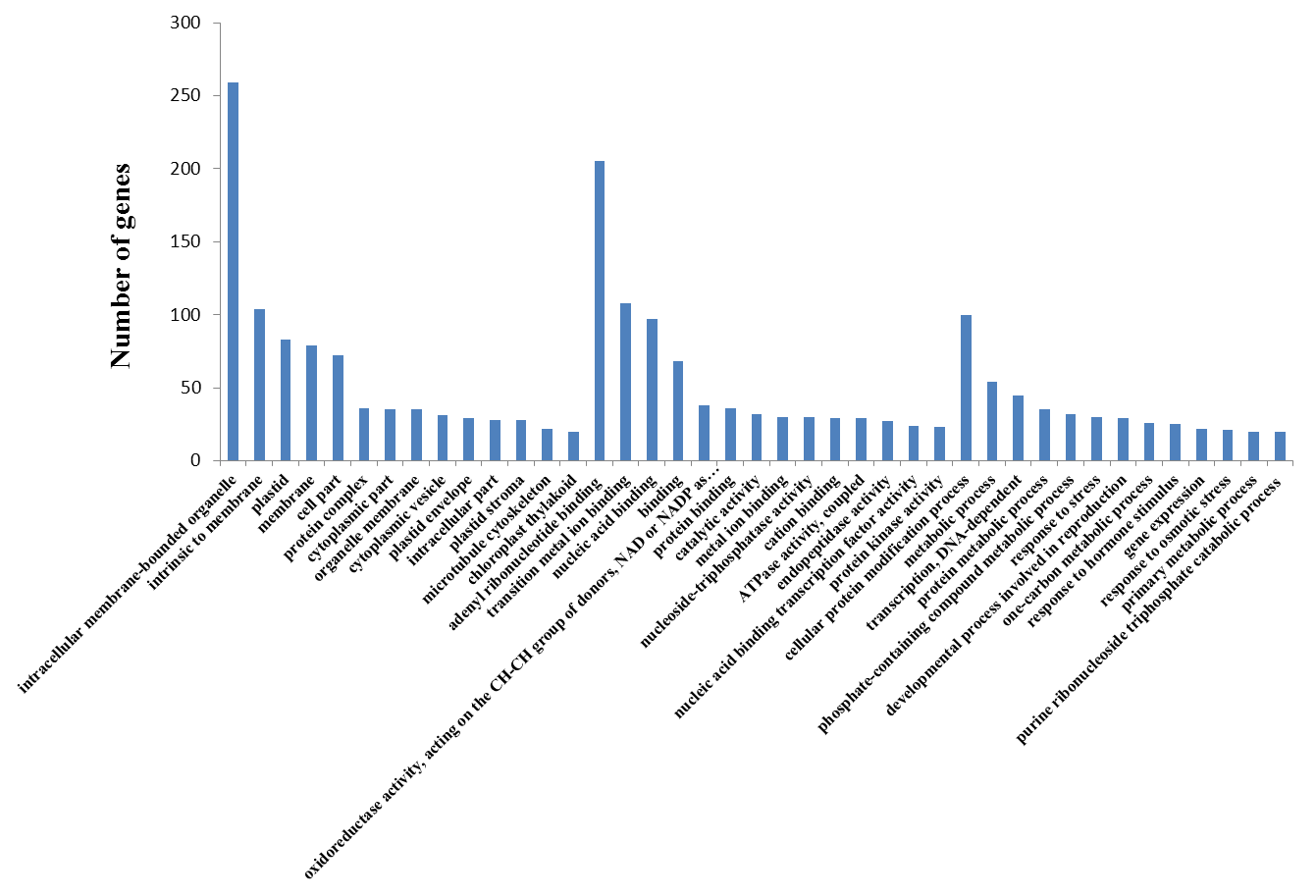


Cellular Component

Molecular Function

Biological Process

**ABML1 VS RML**

**Gene ontology classification of ABML1 VS RML.** Only the main categories (the number of genes in the category ≥ 20) were listed; other 75 sorts including 211 genes in cellular component, other 276 classifications containing 901 genes in molecular function and 431 categories comprised by 1045 genes in biological process were not shown.


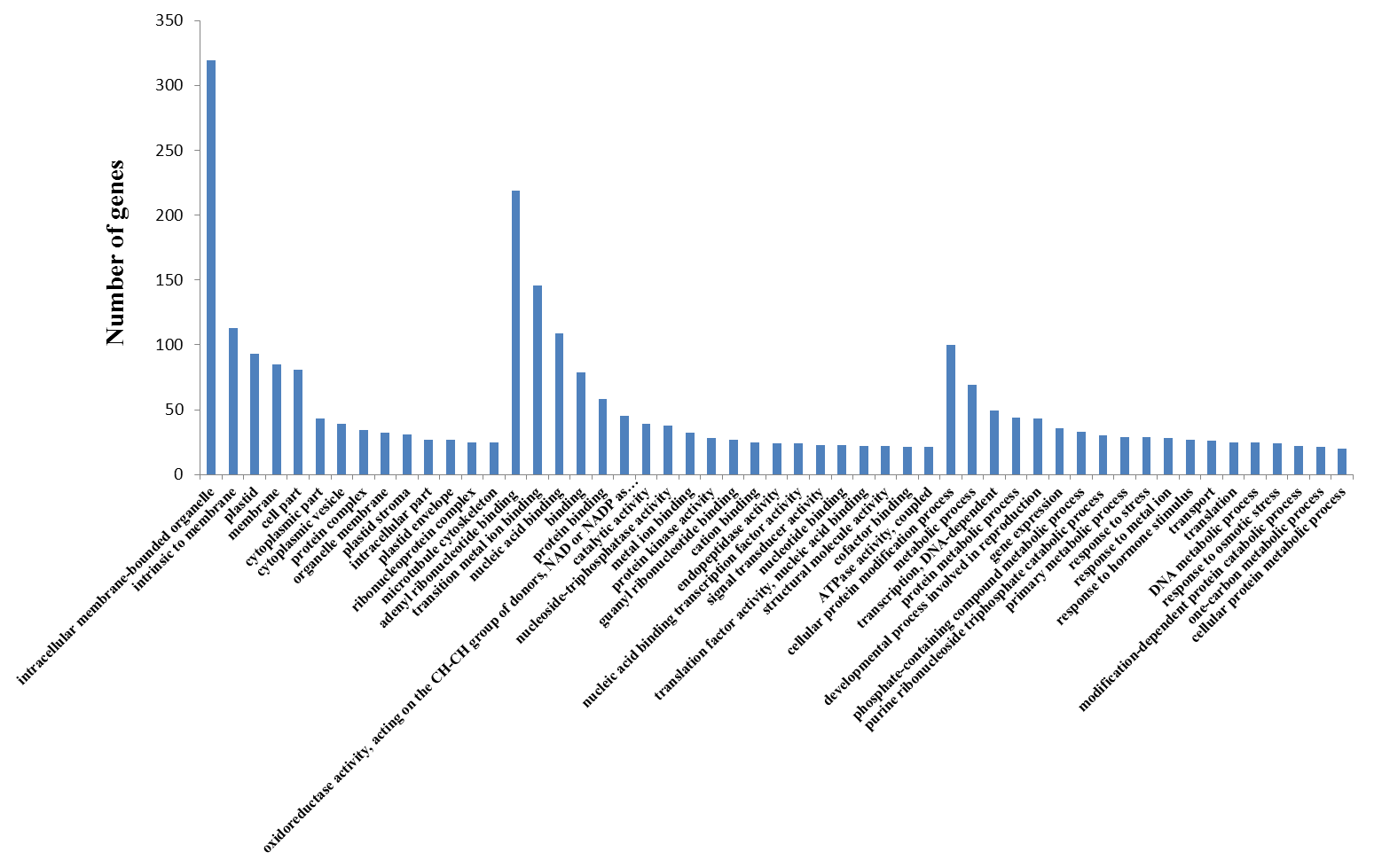


Cellular Component

Molecular Function

Biological Process

**ABPL1 VS ABPL2**

**Gene ontology classification of ABPL1 VS ABPL2.** Only the main categories (the number of genes in the category ≥ 20) were listed; other 97 sorts including 316 genes in cellular component, other 303 classifications containing 934 genes in molecular function and 478 categories comprised by 1181 genes in biological process were not shown.


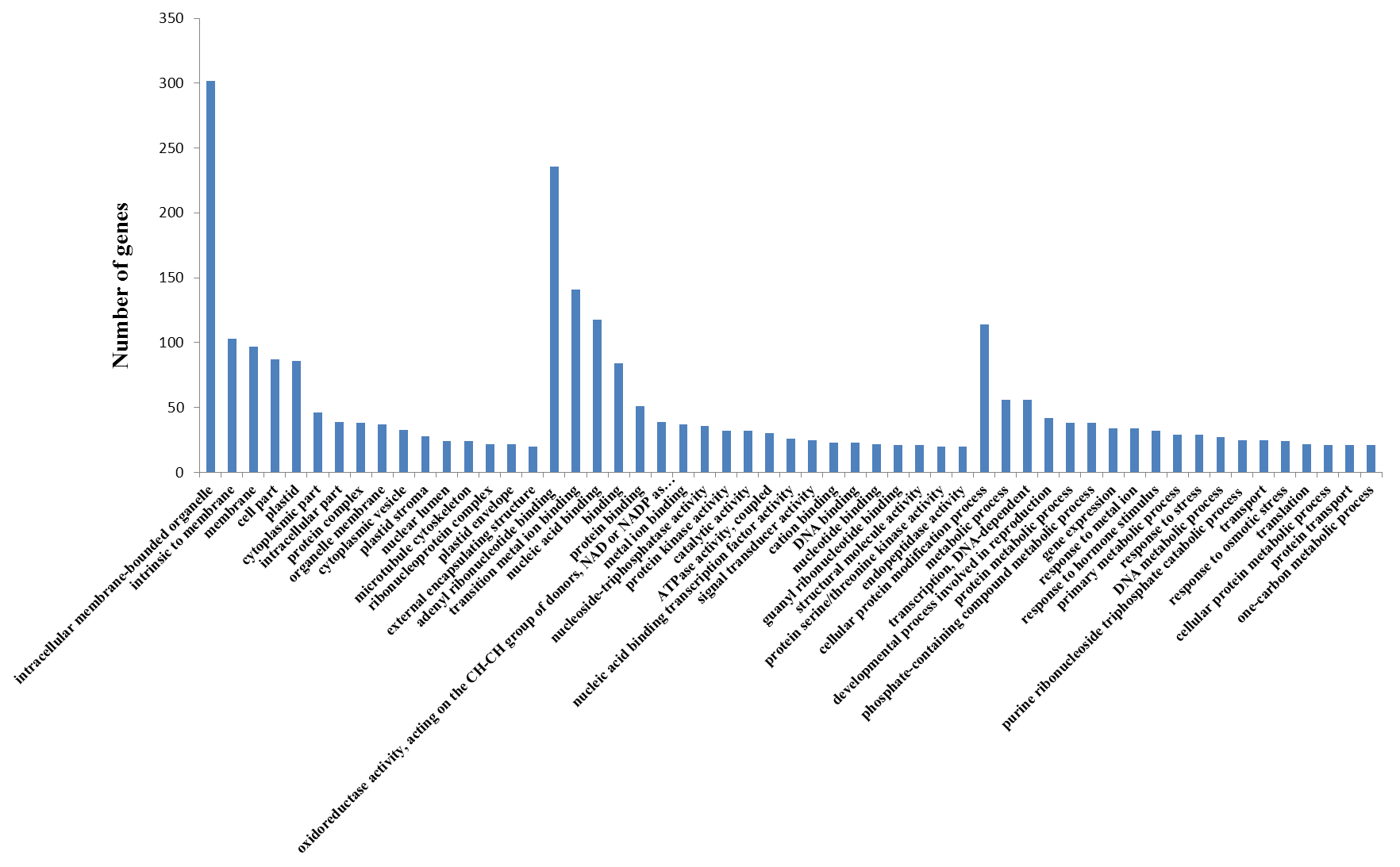


Cellular Component

Molecular Function

Biological Process

**ABPL2 VS RPL**

**Gene ontology classification of ABPL2 VS RPL.** Only the main categories (the number of genes in the category ≥ 20) were listed; other 90 sorts including 246 genes in cellular component, other 304 classifications containing 945 genes in molecular function and 465 categories comprised by 1103 genes in biological process were omitted..


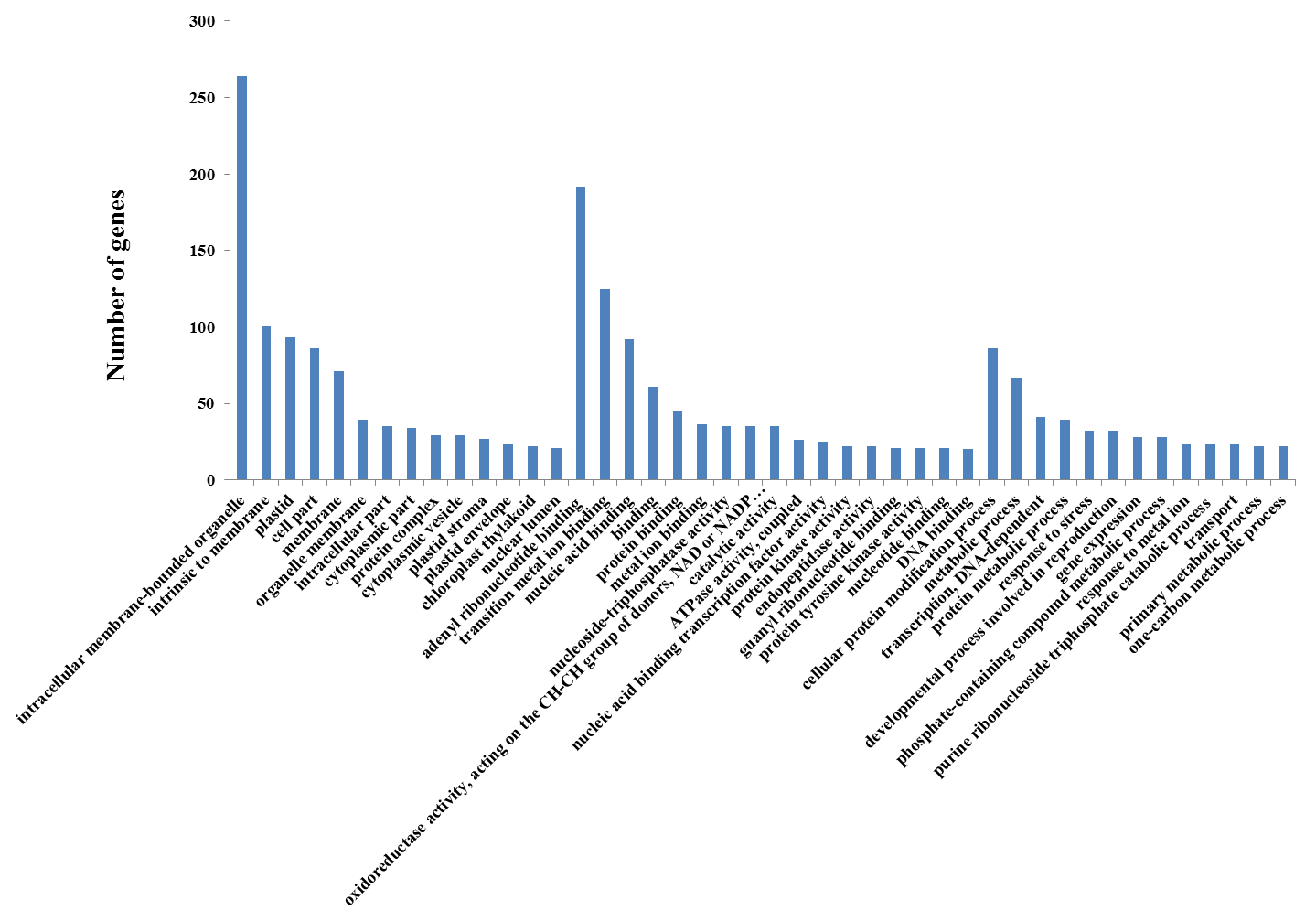


Cellular Component

Molecular Function

Biological Process

**ABPL1 VS RPL**

**Gene ontology classification of ABPL1 VS RPL.** Only the main categories (the number of genes in the category ≥ 20) were listed; other 84 sorts including 254 genes in cellular component, other 309 classifications containing 915 genes in molecular function and 450 categories comprised by 1085 genes in biological process were not shown.


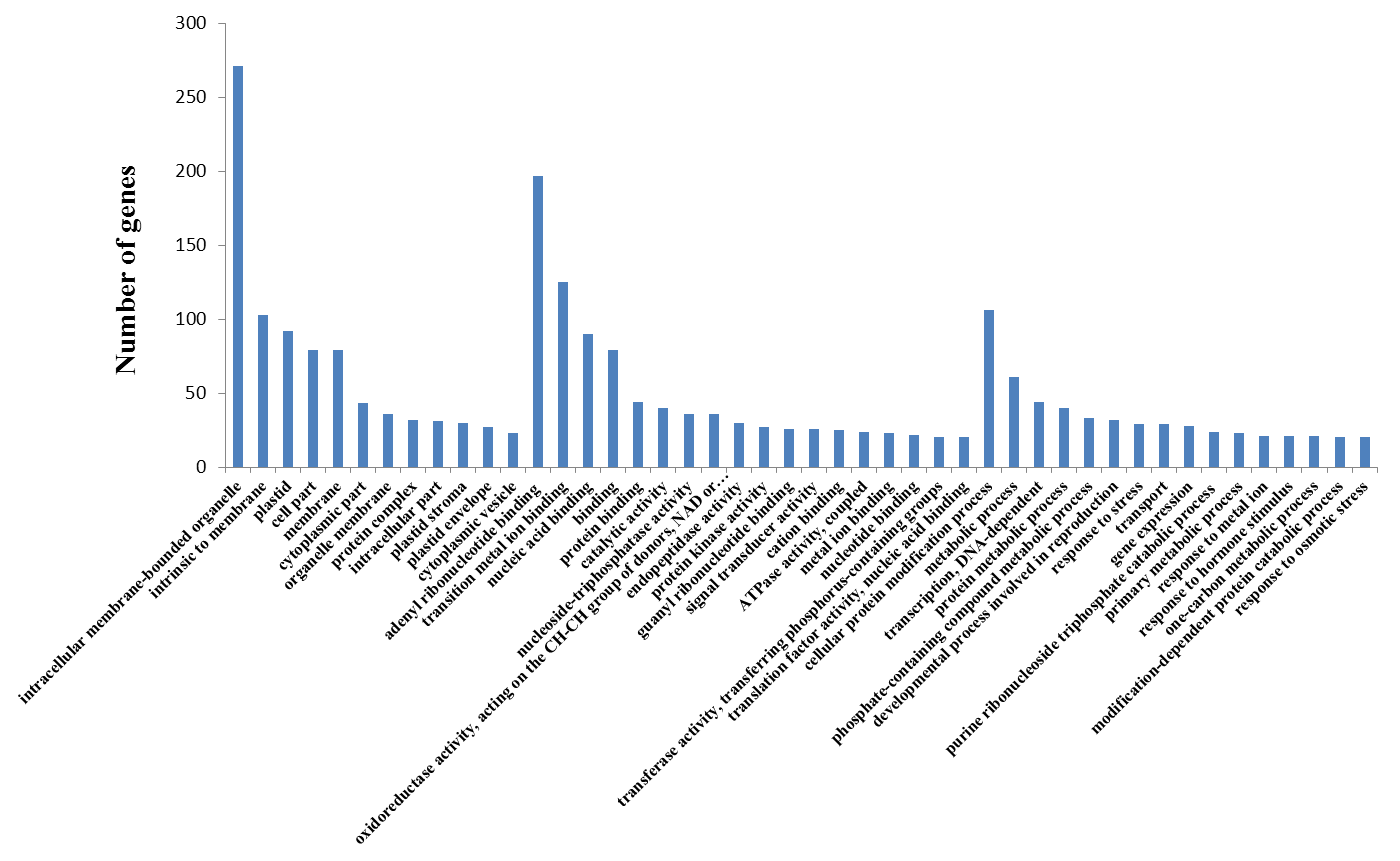


Cellular Component

Molecular Function

Biological Process

**ABML1 VS ABPL1**

**Gene ontology classification of ABML1 VS ABPL1.** Only the main categories (the number of genes in the category ≥ 20) were listed; other 87 sorts including 314 genes in cellular component, other 279 classifications containing 856 genes in molecular function and 424 categories comprised by 1006 genes in biological process were not shown.


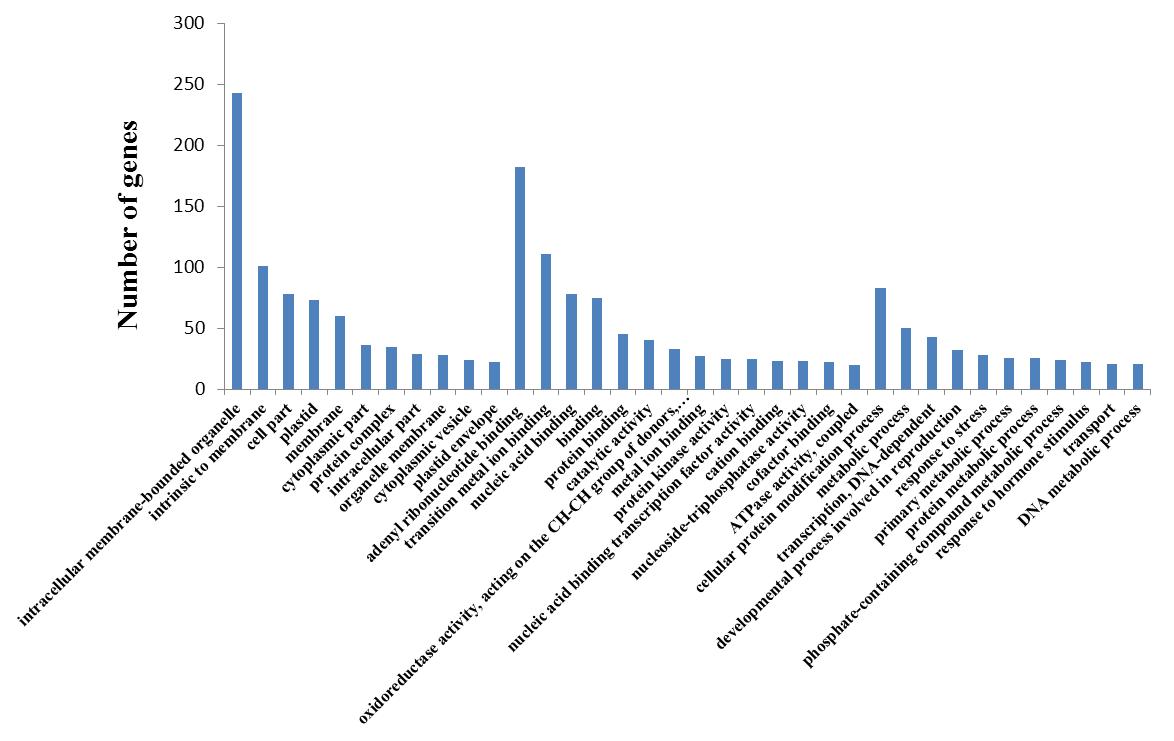


Cellular Component

Molecular Function

Biological Process

**ABML2 VS ABPL2**

**Gene ontology classification of ABML2 VS ABPL2.** Only the main categories (the number of genes in the category ≥ 20) were listed; other 92 sorts including 311 genes in cellular component, other 288 classifications containing 896 genes in molecular function and 444 categories comprised by 1087 genes in biological process were not shown.


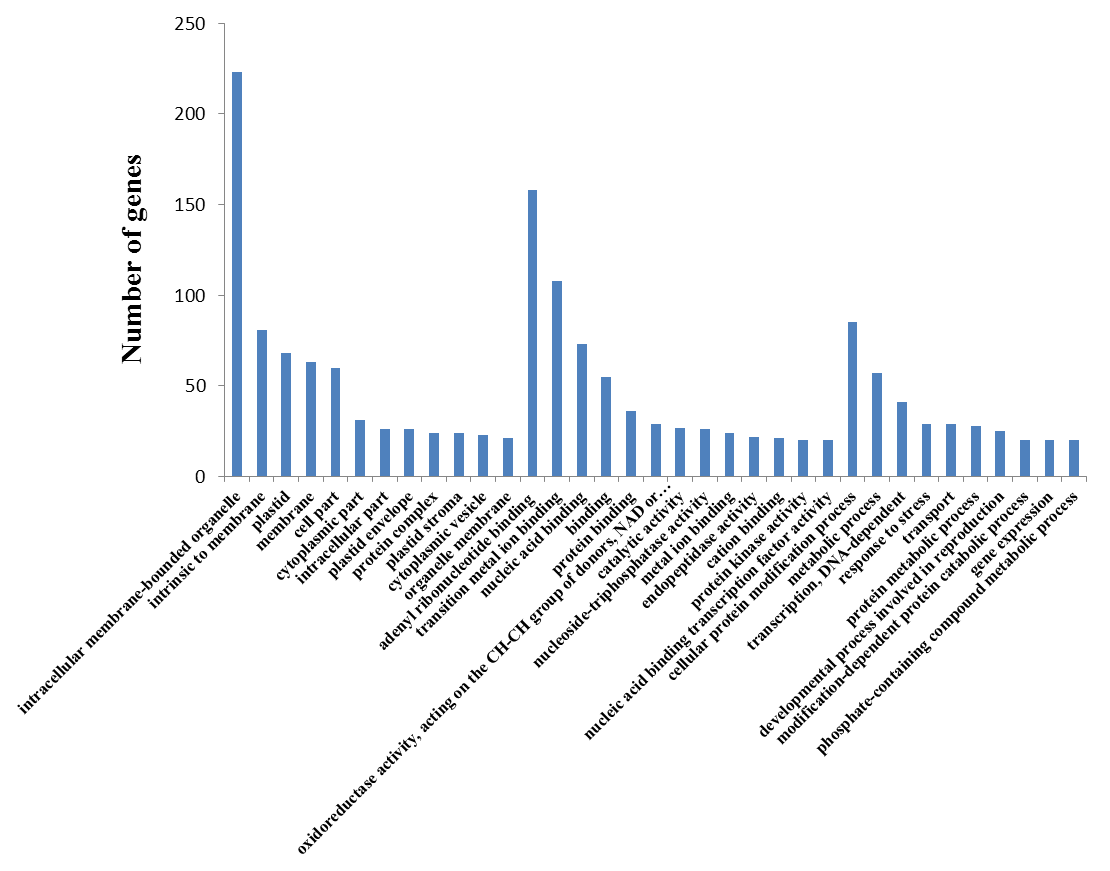


Cellular Component

Molecular Function

Biological Process

**RML VS RPL**

**Gene ontology classification of RML VS RPL.** Only the main categories (the number of genes in the category ≥ 20) were listed; other 73 sorts including 229 genes in cellular component, other 261 classifications containing 793 genes in molecular function and 405 categories comprised by 942 genes in biological process were not shown.
